# Supplementary figures and images for: The photosystem I supercomplex from a primordial green alga Ostreococcus tauri harbors three light-harvesting complex trimers
Source: eLife. 2023 Mar 23;12:e84488. doi: 10.7554/eLife.84488 (PMC10097422; doi:10.7554/eLife.84488)

A1 A3 A3L A4 M

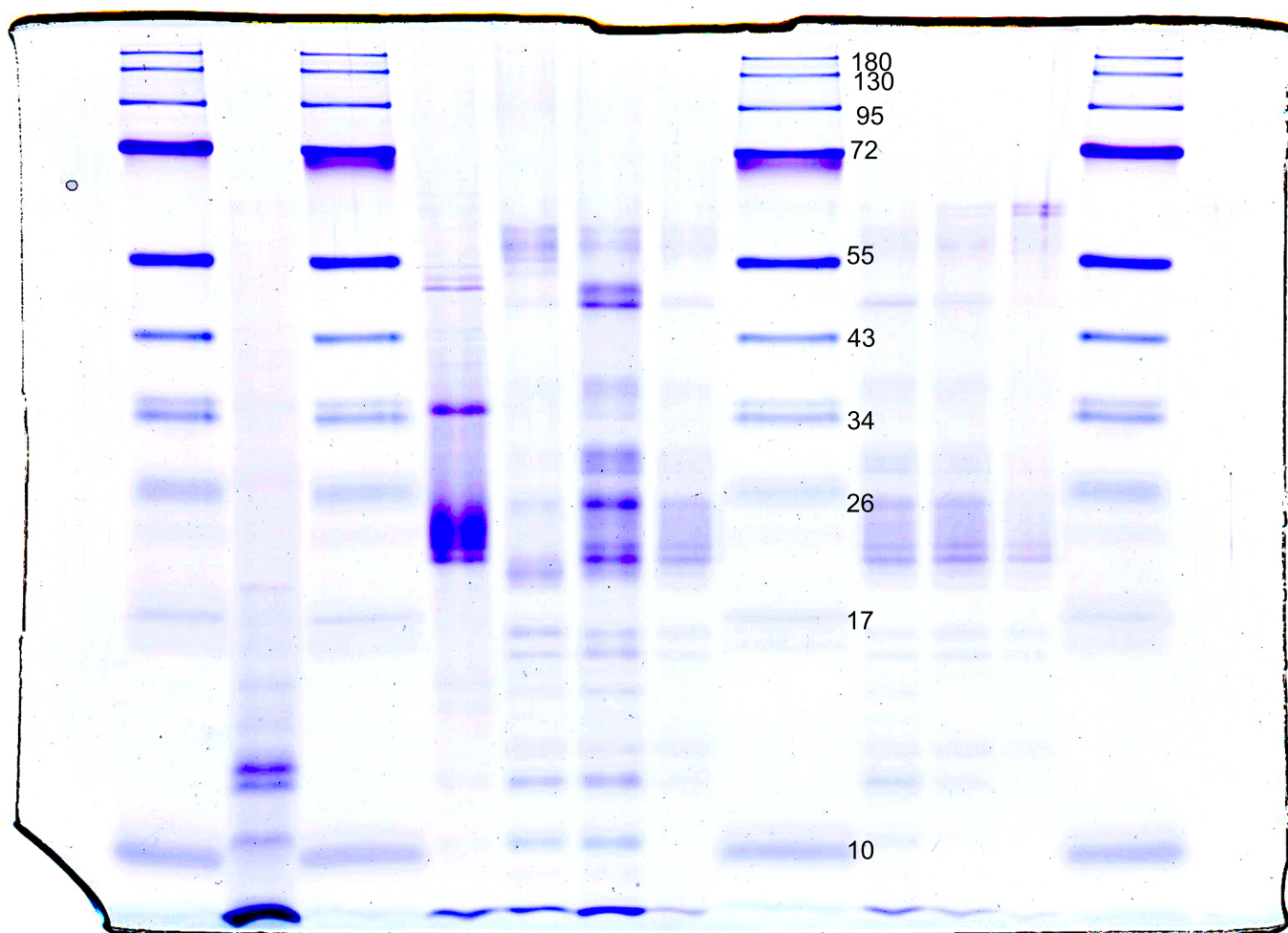

Supplement: Figure 1—figure supplement 1—source data 1. [file elife-84488-fig1-figsupp1-data1.zip › Figure1-figure supplement 1-source data 1/Fig1-figS1b uncropped gel with label/Fig1-FigS1c_edit.pdf]

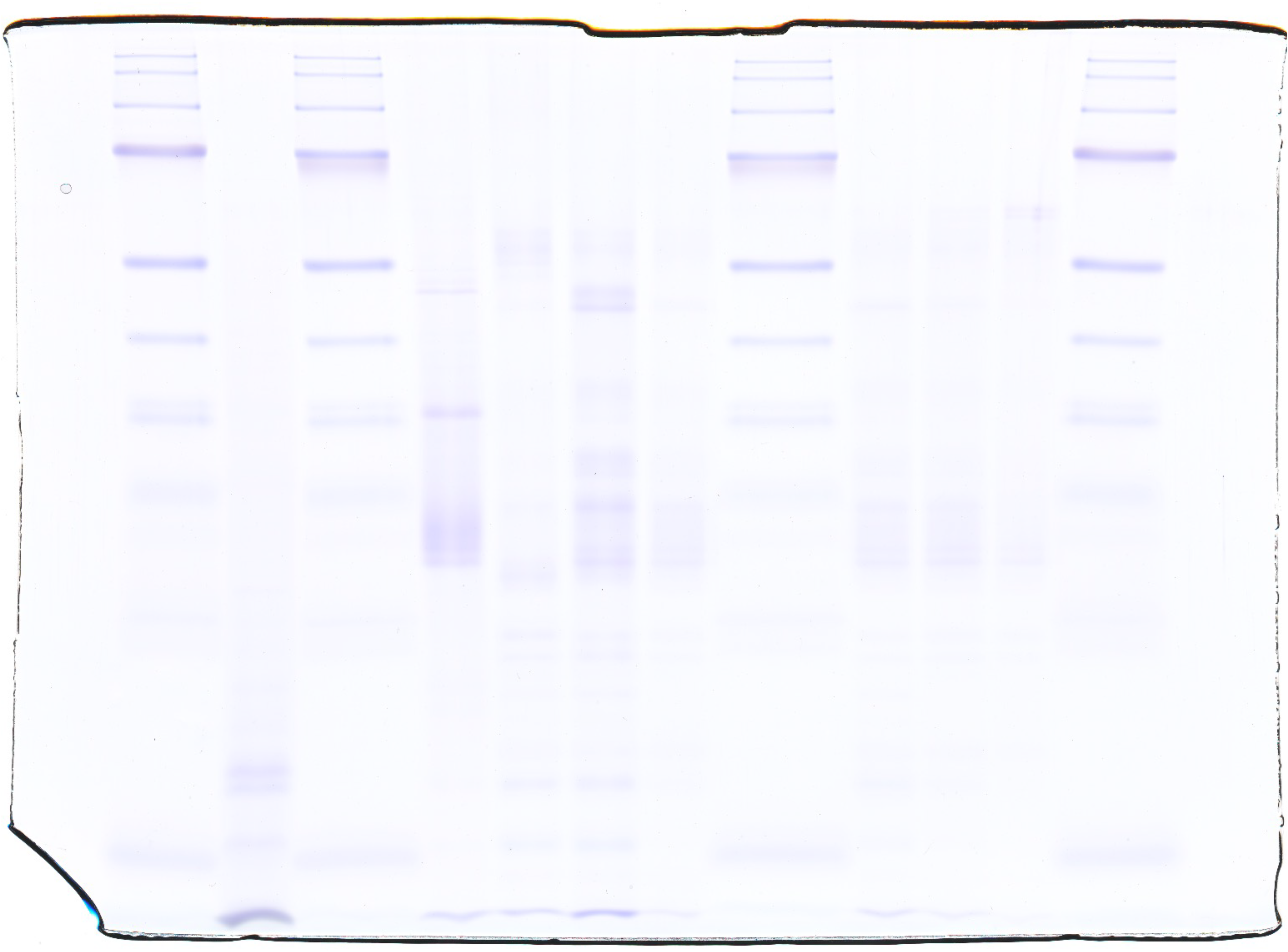

Supplement: Figure 1—figure supplement 1—source data 1. [file elife-84488-fig1-figsupp1-data1.zip › Figure1-figure supplement 1-source data 1/Fig1-figS1b-raw data/Fig1-FigS1c-raw.tif]

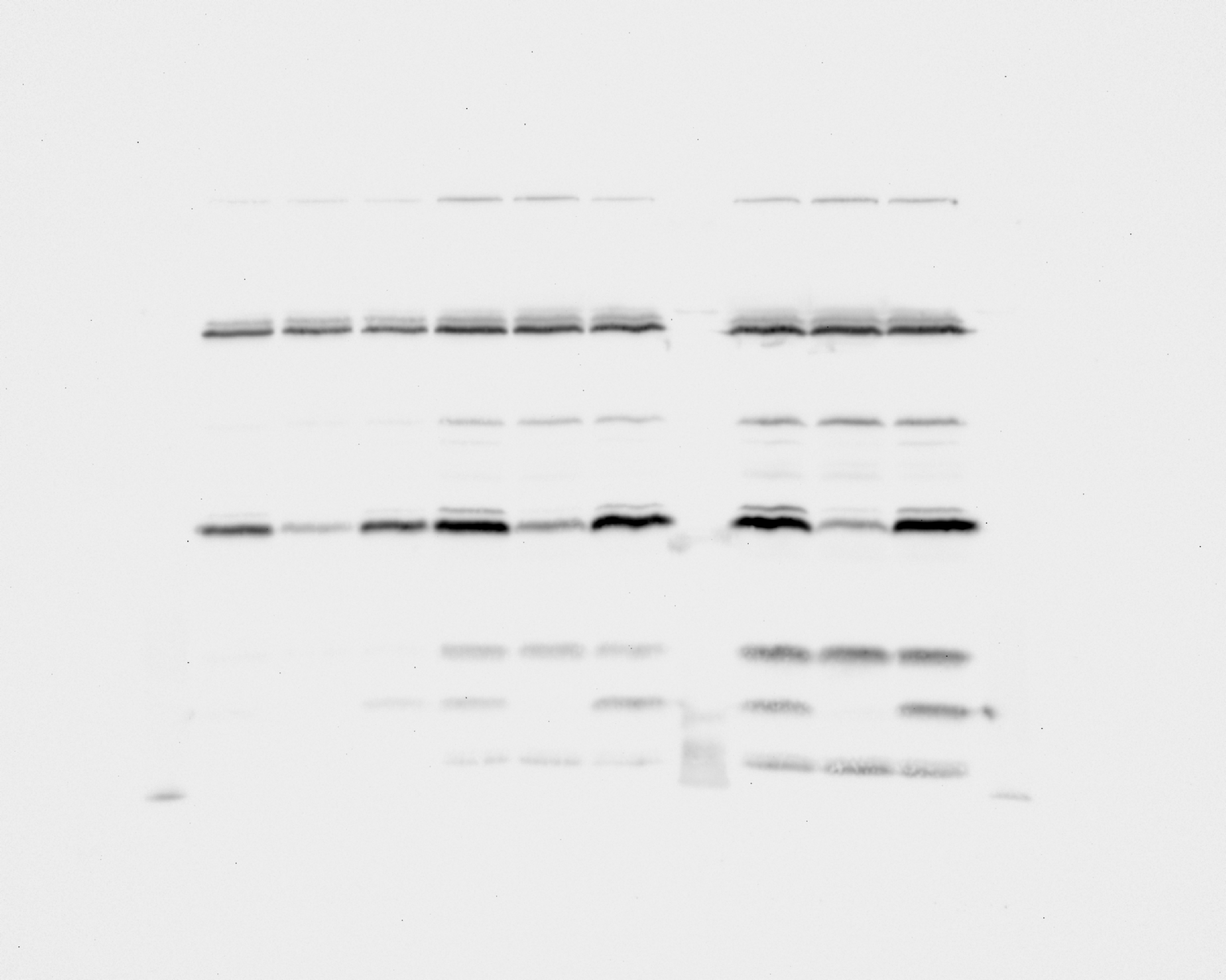

Supplement: Figure 11—source data 2. [file elife-84488-fig11-data2.zip › Figure11-source data 2/Fig 11b raw data/immunoblot1_atpb_lhcp1.tif]

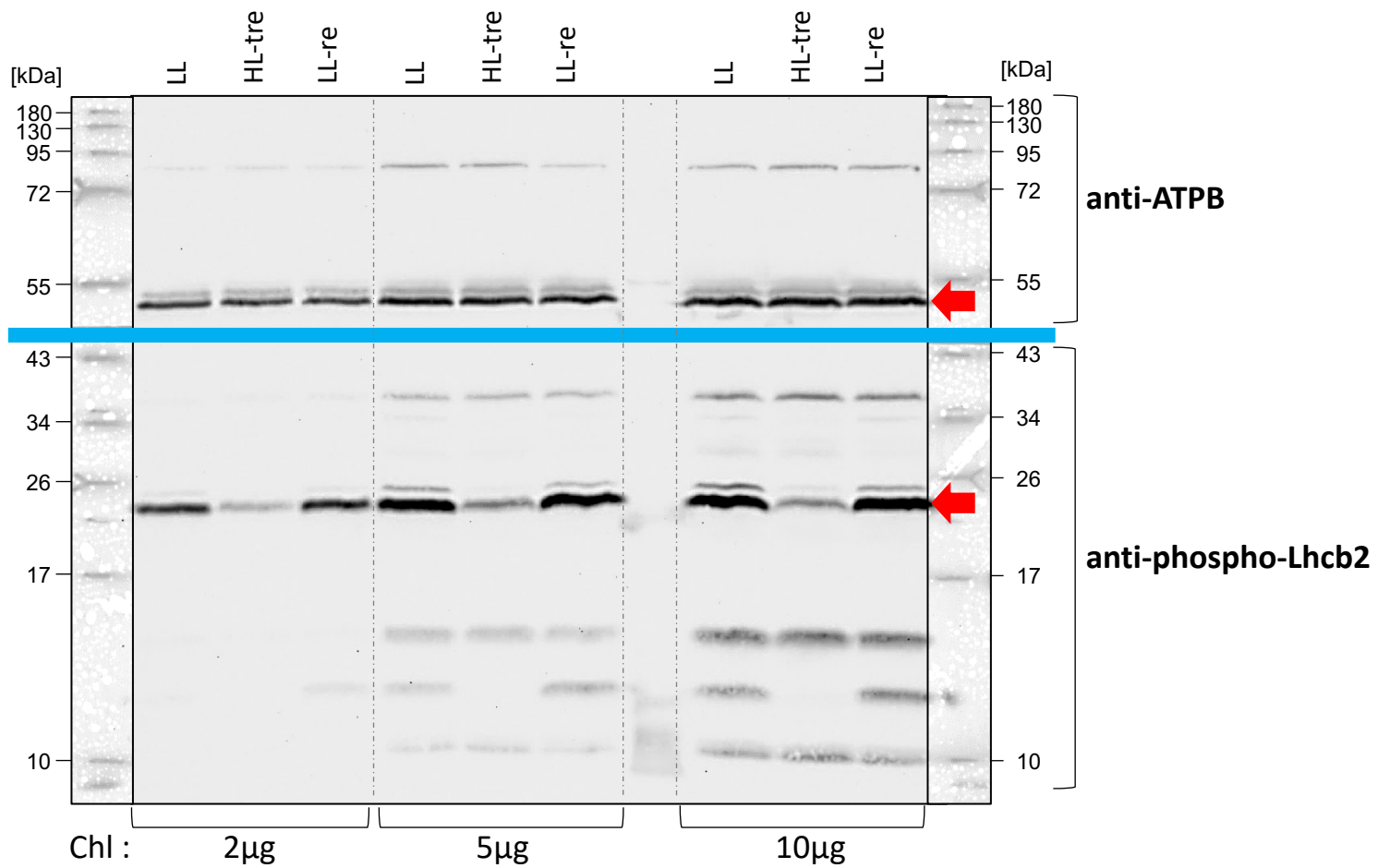

Supplement: Figure 11—source data 2. [file elife-84488-fig11-data2.zip › Figure11-source data 2/Fig 11b uncropped blots with label/immunoblot1_atpb_lhcp1_labeled.pdf]

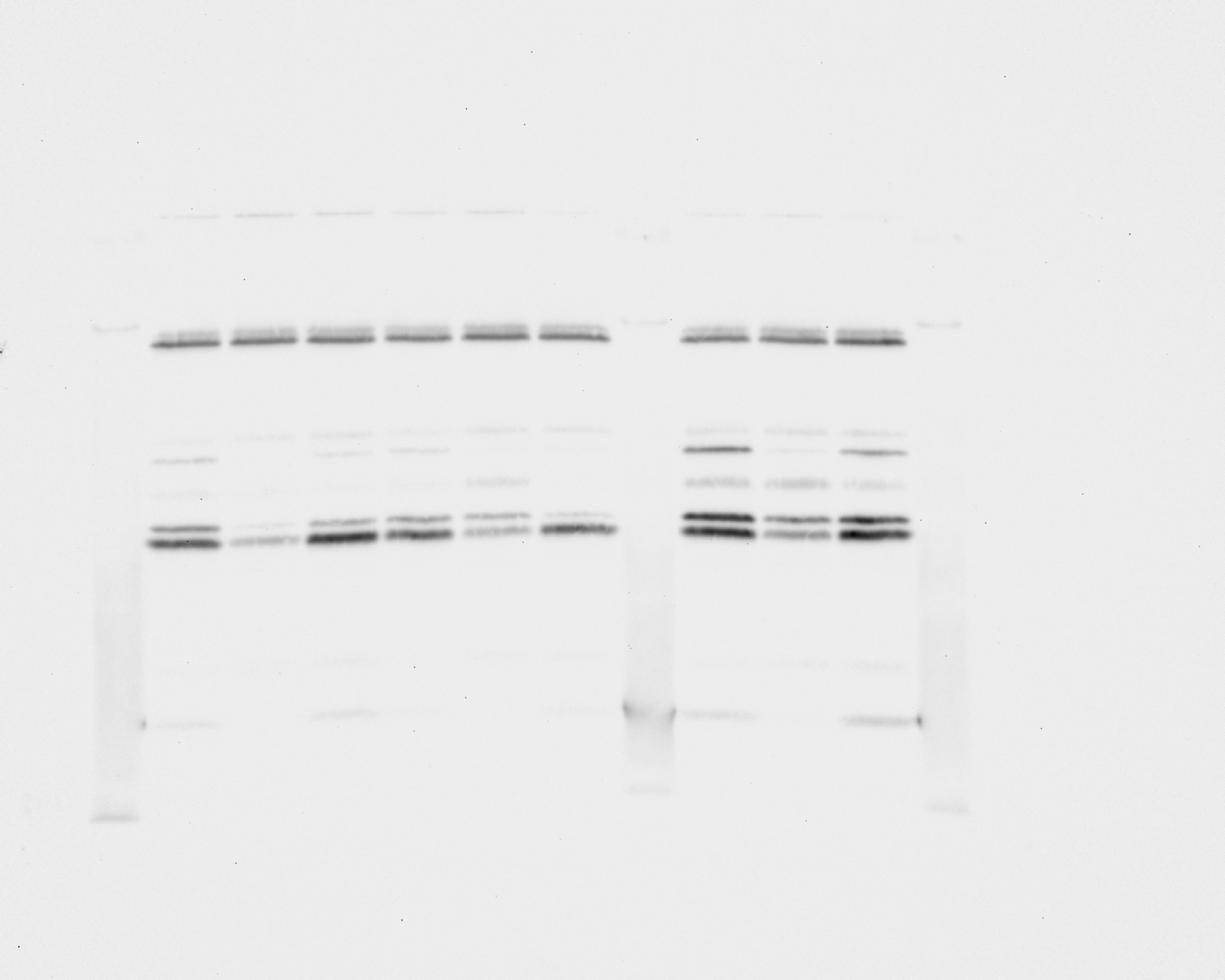

Supplement: Figure 11—figure supplement 1—source data 1. [file elife-84488-fig11-figsupp1-data1.zip › Figure11-figure supplement 1-source data 1/Fig.11_FigS1 raw data/immunoblot2_atpb_lhcp1.tif]

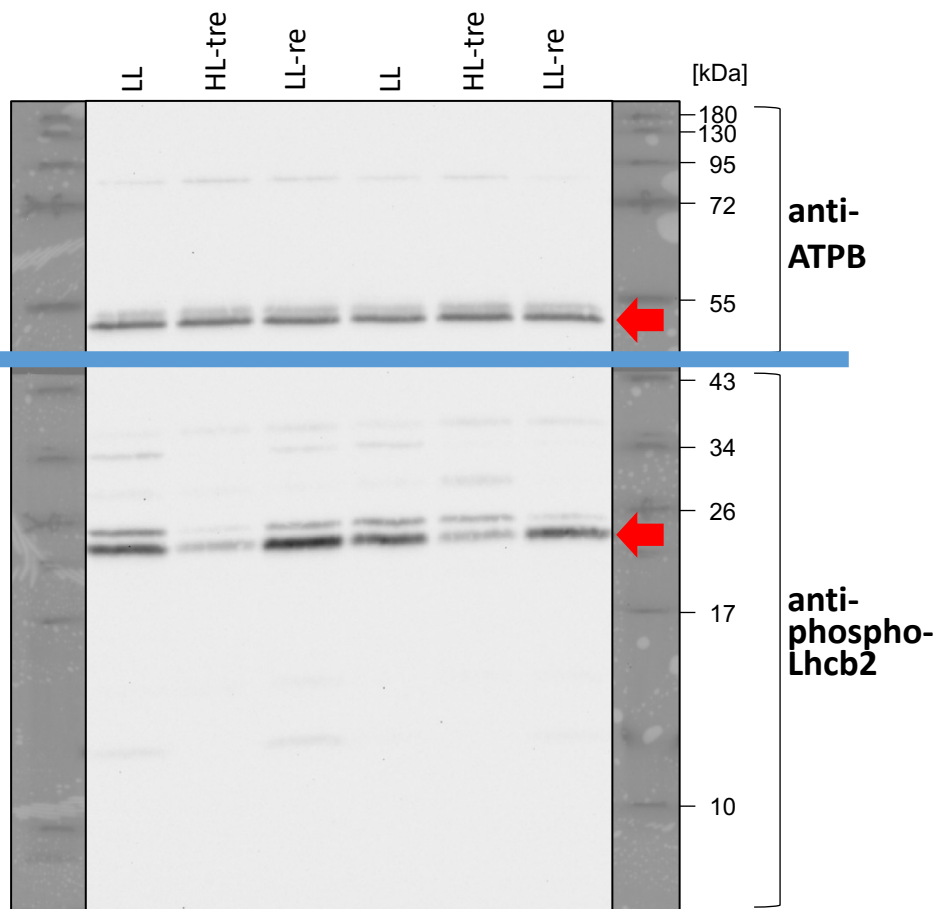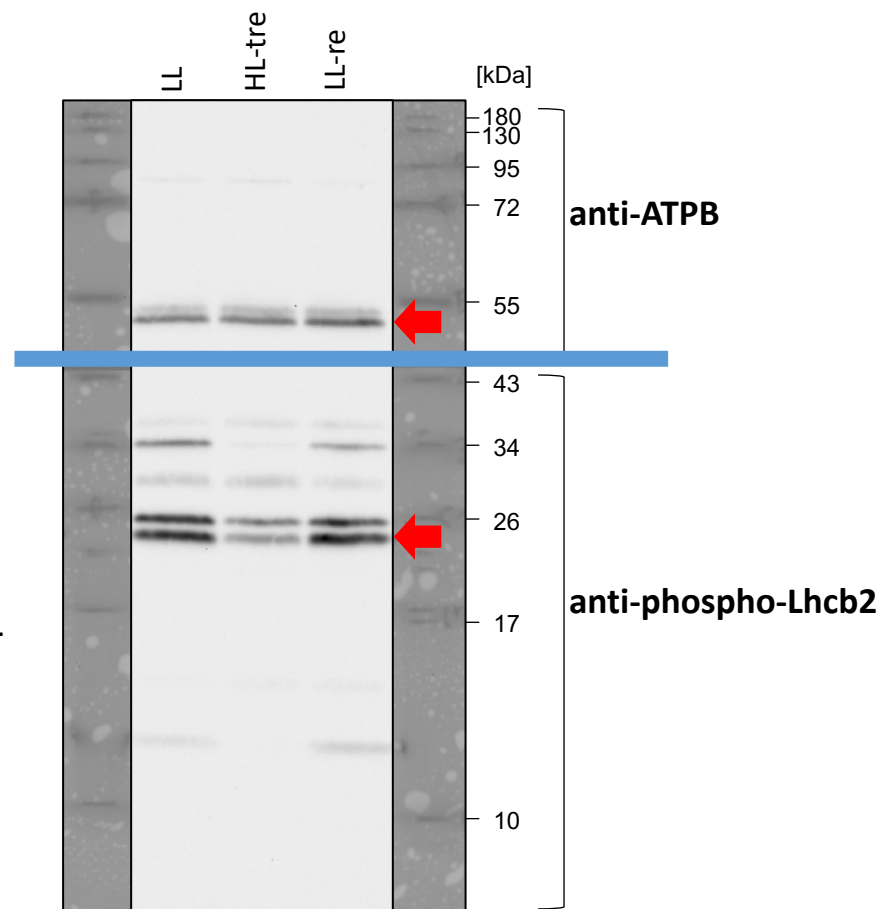

Supplement: Figure 11—figure supplement 1—source data 1. [file elife-84488-fig11-figsupp1-data1.zip › Figure11-figure supplement 1-source data 1/Fig.11_FigS1 uncropped blots with label/immunoblot2_atpb_lhcp1_labeled.pdf]
