# Supplementary material for: The photosystem I supercomplex from a primordial green alga Ostreococcus tauri harbors three light-harvesting complex trimers
Source: eLife. 2023 Mar 23;12:e84488. doi: 10.7554/eLife.84488 (PMC10097422; doi:10.7554/eLife.84488)
Supplement: Supplementary file 1. — (a) Polypeptides in the A3L fraction (PSI-LHCI-Lhcp supercomplex) as identified by MS analysis. The peptides were evaluated using liquid chomatography tandem mass spectrometry analysis. Detected peptides were analyzed using Mascot ver.2.7.0 (Matrix Science, London W1U 7GB, UK) and Proteome Discoverer software (Thermo Fisher Scientific). Observed: Experimental m/z value, Mr(expet): Experimental m/z transformed to a relative molecular mass, Mr(calc): Relative molecular mass calculated from the matched peptide sequence, Score: The ions score is a value of matching level between product ion peak and calculated fragment by MASCOT. (b) Polypeptides in the A3 fraction (PSI-LHCI supercomplex) as identified by MS analysis. The bands corresponding to Lhca5 and Lhca6 (Bands 2 and 1, respectively) were excised from the SDS-PAGE gel and subsequently subjected to LC-MS/MS analysis following in-gel trypsin digestion, as described in Kubota-Kawai et al., 2019. Detected peptides were analyzed as in (a). [file elife-84488-supp1.docx]

**Supplementary File 1a. Polypeptides in the A3L fraction (PSI-LHCI-Lhcp supercomplex) as identified by MS analysis.** The peptides were evaluated using liquid chomatography tandem mass spectrometry analysis. Detected peptides were analyzed using Mascot ver.2.7.0 (Matrix science, London W1U 7GB, UK) and Proteome Discoverer software (Thermo Fisher Scientific). Observed: Experimental m/z value, Mr(expet): Experimental m/z transformed to a relative molecular mass, Mr(calc): Relative molecular mass calculated from the matched peptide sequence, Score: The ions score is a value of matching level between product ion peak and calculated fragment by MASCOT.

| PsaA | | | | | | |
| --- | --- | --- | --- | --- | --- | --- |
| Observed | Mr(expt) | Mr(calc) | Score | Peptide sequence | Amino acid position  start | Amino acid position  end |
| 801.4304 | 1600.8462 | 1600.8461 | 48 | IVVDRDPVPTSFEK | 15 | 28 |
| 534.6227 | 1600.8462 | 1600.8461 | 21 | IVVDRDPVPTSFEK | 15 | 28 |
| 534.6229 | 1600.8468 | 1600.8461 | 22 | IVVDRDPVPTSFEK | 15 | 28 |
| 801.431 | 1600.8475 | 1600.8461 | 63 | IVVDRDPVPTSFEK | 15 | 28 |
| 801.433 | 1600.8515 | 1600.8461 | 40 | IVVDRDPVPTSFEK | 15 | 28 |
| 510.2565 | 1018.4984 | 1018.4971 | 17 | DPVPTSFEK | 20 | 28 |
| 543.2858 | 1084.557 | 1084.5566 | 33 | WAKPGHFSR | 29 | 37 |
| 748.3909 | 1494.7672 | 1494.7653 | 41 | EIPLPHEFMLNR | 236 | 247 |
| 504.5939 | 1510.7599 | 1510.7602 | 23 | EIPLPHEFMLNR | 236 | 247 |
| 670.8488 | 1339.6829 | 1339.6846 | 34 | ALMAELYPSFAK | 248 | 259 |
| 670.849 | 1339.6834 | 1339.6846 | 38 | ALMAELYPSFAK | 248 | 259 |
| 670.8492 | 1339.6839 | 1339.6846 | 40 | ALMAELYPSFAK | 248 | 259 |
| 670.8493 | 1339.684 | 1339.6846 | 38 | ALMAELYPSFAK | 248 | 259 |
| 670.8497 | 1339.6848 | 1339.6846 | 41 | ALMAELYPSFAK | 248 | 259 |
| 670.8497 | 1339.6849 | 1339.6846 | 37 | ALMAELYPSFAK | 248 | 259 |
| 670.8498 | 1339.685 | 1339.6846 | 50 | ALMAELYPSFAK | 248 | 259 |
| 670.8498 | 1339.6851 | 1339.6846 | 45 | ALMAELYPSFAK | 248 | 259 |
| 670.8499 | 1339.6853 | 1339.6846 | 36 | ALMAELYPSFAK | 248 | 259 |
| 670.8499 | 1339.6853 | 1339.6846 | 60 | ALMAELYPSFAK | 248 | 259 |
| 670.85 | 1339.6854 | 1339.6846 | 55 | ALMAELYPSFAK | 248 | 259 |
| 670.85 | 1339.6855 | 1339.6846 | 28 | ALMAELYPSFAK | 248 | 259 |
| 670.8502 | 1339.6858 | 1339.6846 | 45 | ALMAELYPSFAK | 248 | 259 |
| 670.8504 | 1339.6862 | 1339.6846 | 25 | ALMAELYPSFAK | 248 | 259 |
| 670.8511 | 1339.6876 | 1339.6846 | 32 | ALMAELYPSFAK | 248 | 259 |
| 678.8463 | 1355.6781 | 1355.6795 | 63 | ALMAELYPSFAK | 248 | 259 |
| 678.8463 | 1355.6781 | 1355.6795 | 55 | ALMAELYPSFAK | 248 | 259 |
| 678.8466 | 1355.6787 | 1355.6795 | 50 | ALMAELYPSFAK | 248 | 259 |
| 678.8472 | 1355.6799 | 1355.6795 | 66 | ALMAELYPSFAK | 248 | 259 |
| 678.8475 | 1355.6804 | 1355.6795 | 57 | ALMAELYPSFAK | 248 | 259 |
| 678.8476 | 1355.6806 | 1355.6795 | 63 | ALMAELYPSFAK | 248 | 259 |
| 377.5189 | 1129.5348 | 1129.5339 | 34 | TNWGIGHSMK | 314 | 323 |
| 565.7753 | 1129.536 | 1129.5339 | 33 | TNWGIGHSMK | 314 | 323 |
| 573.7724 | 1145.5302 | 1145.5288 | 40 | TNWGIGHSMK | 314 | 323 |
| 420.2346 | 838.4547 | 838.4548 | 19 | EILEAHK | 324 | 330 |
| 875.4508 | 1748.8871 | 1748.8846 | 75 | EILEAHKGPFTGEGHK | 324 | 339 |
| 583.9698 | 1748.8875 | 1748.8846 | 24 | EILEAHKGPFTGEGHK | 324 | 339 |
| 557.2528 | 1668.7364 | 1668.738 | 45 | DYDPATNYNNVLDR | 416 | 429 |
| 835.3763 | 1668.7381 | 1668.738 | 100 | DYDPATNYNNVLDR | 416 | 429 |
| 835.3766 | 1668.7386 | 1668.738 | 83 | DYDPATNYNNVLDR | 416 | 429 |
| 835.3772 | 1668.7398 | 1668.738 | 68 | DYDPATNYNNVLDR | 416 | 429 |
| 835.3776 | 1668.7407 | 1668.738 | 76 | DYDPATNYNNVLDR | 416 | 429 |
| 835.3782 | 1668.7418 | 1668.738 | 66 | DYDPATNYNNVLDR | 416 | 429 |
| 622.3621 | 1242.7097 | 1242.7084 | 18 | LIPDKANLGFR | 562 | 572 |
| 453.2 | 904.3853 | 904.3862 | 22 | FPCDGPGR | 573 | 580 |
| 453.2004 | 904.3862 | 904.3862 | 39 | FPCDGPGR | 573 | 580 |
| 453.2006 | 904.3867 | 904.3862 | 26 | FPCDGPGR | 573 | 580 |
| 1193.2332 | 3576.6776 | 3576.6805 | 97 | MQSDVWGSVTGNGVSHITGGNFAQSANTINGWLR | 614 | 647 |
| 658.3359 | 1971.9858 | 1971.9843 | 32 | GYWQELIESIVWAHNK | 691 | 706 |
| 987.0013 | 1971.988 | 1971.9843 | 58 | GYWQELIESIVWAHNK | 691 | 706 |
| 426.2583 | 850.502 | 850.5025 | 43 | VAPAIQPR | 709 | 716 |
| 423.2458 | 844.477 | 844.4767 | 39 | ALSITQGR | 717 | 724 |
| 423.2458 | 844.477 | 844.4767 | 39 | ALSITQGR | 717 | 724 |
| 784.0951 | 2349.2634 | 2349.2634 | 43 | AVGVAHYLLGGIATTWSFFLAR | 725 | 746 |

| PsaB | | | | | | |
| --- | --- | --- | --- | --- | --- | --- |
| Observed | Mr(expt) | Mr(calc) | Score | Peptide sequence | Amino acid position  start | Amino acid position  end |
| 640.3151 | 1278.6156 | 1278.6204 | 68 | FSQGLASDPTTR | 8 | 19 |
| 640.3163 | 1278.618 | 1278.6204 | 31 | FSQGLASDPTTR | 8 | 19 |
| 640.3174 | 1278.6203 | 1278.6204 | 56 | FSQGLASDPTTR | 8 | 19 |
| 640.3176 | 1278.6206 | 1278.6204 | 40 | FSQGLASDPTTR | 8 | 19 |
| 640.3179 | 1278.6212 | 1278.6204 | 72 | FSQGLASDPTTR | 8 | 19 |
| 640.3179 | 1278.6213 | 1278.6204 | 55 | FSQGLASDPTTR | 8 | 19 |
| 640.318 | 1278.6215 | 1278.6204 | 46 | FSQGLASDPTTR | 8 | 19 |
| 640.3182 | 1278.6219 | 1278.6204 | 53 | FSQGLASDPTTR | 8 | 19 |
| 479.2484 | 1434.7235 | 1434.7215 | 44 | FSQGLASDPTTRR | 8 | 20 |
| 718.3694 | 1434.7243 | 1434.7215 | 33 | FSQGLASDPTTRR | 8 | 20 |
| 648.5548 | 2590.1901 | 2590.1911 | 33 | RIWFGIATAHDFETHDGMTEEK | 20 | 41 |
| 652.5526 | 2606.1811 | 2606.186 | 46 | RIWFGIATAHDFETHDGMTEEK | 20 | 41 |
| 869.7368 | 2606.1885 | 2606.186 | 42 | RIWFGIATAHDFETHDGMTEEK | 20 | 41 |
| 812.3689 | 2434.0849 | 2434.09 | 53 | IWFGIATAHDFETHDGMTEEK | 21 | 41 |
| 817.7022 | 2450.0848 | 2450.0849 | 47 | IWFGIATAHDFETHDGMTEEK | 21 | 41 |
| 870.6916 | 3478.7375 | 3478.7323 | 39 | WGEDPLHVRPIAHTIWDPHFGQPAVEAFTR | 77 | 106 |
| 1332.141 | 2662.2674 | 2662.2638 | 99 | GGASAPVNIAYSGVYQWWYTIGMR | 107 | 130 |
| 1026.224 | 3075.6502 | 3075.6519 | 76 | LNHHLSGLFGVSSLAWTGHLVHVAIPASR | 175 | 203 |
| 616.1376 | 3075.6518 | 3075.6519 | 57 | LNHHLSGLFGVSSLAWTGHLVHVAIPASR | 175 | 203 |
| 769.9206 | 3075.6533 | 3075.6519 | 67 | LNHHLSGLFGVSSLAWTGHLVHVAIPASR | 175 | 203 |
| 513.6165 | 3075.6555 | 3075.6519 | 32 | LNHHLSGLFGVSSLAWTGHLVHVAIPASR | 175 | 203 |
| 364.5152 | 1090.5237 | 1090.523 | 22 | TNFGIGHSMK | 293 | 302 |
| 546.2696 | 1090.5246 | 1090.523 | 36 | TNFGIGHSMK | 293 | 302 |
| 554.2649 | 1106.5152 | 1106.5179 | 24 | TNFGIGHSMK | 293 | 302 |
| 369.846 | 1106.5163 | 1106.5179 | 25 | TNFGIGHSMK | 293 | 302 |
| 369.8462 | 1106.5167 | 1106.5179 | 21 | TNFGIGHSMK | 293 | 302 |
| 554.2665 | 1106.5185 | 1106.5179 | 42 | TNFGIGHSMK | 293 | 302 |
| 554.2673 | 1106.52 | 1106.5179 | 22 | TNFGIGHSMK | 293 | 302 |
| 554.2678 | 1106.5211 | 1106.5179 | 20 | TNFGIGHSMK | 293 | 302 |
| 427.5578 | 1279.6516 | 1279.652 | 31 | EILEAHTAPSGR | 303 | 314 |
| 640.8331 | 1279.6517 | 1279.652 | 31 | EILEAHTAPSGR | 303 | 314 |
| 640.8334 | 1279.6522 | 1279.652 | 74 | EILEAHTAPSGR | 303 | 314 |
| 476.2066 | 950.3986 | 950.3981 | 30 | DYDPEANK | 397 | 404 |
| 781.3823 | 1560.7501 | 1560.7532 | 83 | DYDPEANKGNVLAR | 397 | 410 |
| 521.258 | 1560.7522 | 1560.7532 | 24 | DYDPEANKGNVLAR | 397 | 410 |
| 781.385 | 1560.7555 | 1560.7532 | 28 | DYDPEANKGNVLAR | 397 | 410 |
| 521.2597 | 1560.7573 | 1560.7532 | 29 | DYDPEANKGNVLAR | 397 | 410 |
| 680.7132 | 2039.1178 | 2039.1204 | 50 | QILIEPVFAQWIQAAQGK | 452 | 469 |
| 1020.567 | 2039.1194 | 2039.1204 | 92 | QILIEPVFAQWIQAAQGK | 452 | 469 |
| 680.7138 | 2039.1196 | 2039.1204 | 48 | QILIEPVFAQWIQAAQGK | 452 | 469 |
| 680.714 | 2039.1201 | 2039.1204 | 69 | QILIEPVFAQWIQAAQGK | 452 | 469 |
| 680.714 | 2039.1203 | 2039.1204 | 51 | QILIEPVFAQWIQAAQGK | 452 | 469 |
| 680.714 | 2039.1203 | 2039.1204 | 56 | QILIEPVFAQWIQAAQGK | 452 | 469 |
| 1020.5682 | 2039.1219 | 2039.1204 | 95 | QILIEPVFAQWIQAAQGK | 452 | 469 |
| 680.7146 | 2039.122 | 2039.1204 | 62 | QILIEPVFAQWIQAAQGK | 452 | 469 |
| 1020.5684 | 2039.1222 | 2039.1204 | 79 | QILIEPVFAQWIQAAQGK | 452 | 469 |
| 1020.5685 | 2039.1224 | 2039.1204 | 86 | QILIEPVFAQWIQAAQGK | 452 | 469 |
| 1020.5687 | 2039.1229 | 2039.1204 | 81 | QILIEPVFAQWIQAAQGK | 452 | 469 |
| 680.715 | 2039.1233 | 2039.1204 | 49 | QILIEPVFAQWIQAAQGK | 452 | 469 |
| 680.715 | 2039.1233 | 2039.1204 | 59 | QILIEPVFAQWIQAAQGK | 452 | 469 |
| 1020.5689 | 2039.1233 | 2039.1204 | 91 | QILIEPVFAQWIQAAQGK | 452 | 469 |
| 680.7152 | 2039.1236 | 2039.1204 | 58 | QILIEPVFAQWIQAAQGK | 452 | 469 |
| 1020.5696 | 2039.1246 | 2039.1204 | 77 | QILIEPVFAQWIQAAQGK | 452 | 469 |
| 1020.5704 | 2039.1262 | 2039.1204 | 77 | QILIEPVFAQWIQAAQGK | 452 | 469 |
| 1020.5709 | 2039.1272 | 2039.1204 | 91 | QILIEPVFAQWIQAAQGK | 452 | 469 |
| 737.8068 | 1473.599 | 1473.5983 | 55 | DFGYSFPCDGPGR | 551 | 563 |
| 737.8077 | 1473.6008 | 1473.5983 | 44 | DFGYSFPCDGPGR | 551 | 563 |
| 942.7823 | 2825.325 | 2825.3231 | 59 | HLALWQGNSAQFDESSTYIMGWLR | 597 | 620 |
| 445.2608 | 888.507 | 888.5069 | 40 | TPLANFVK | 684 | 691 |
| 445.2611 | 888.5077 | 888.5069 | 41 | TPLANFVK | 684 | 691 |
| 445.2612 | 888.5079 | 888.5069 | 36 | TPLANFVK | 684 | 691 |
| 445.2614 | 888.5081 | 888.5069 | 48 | TPLANFVK | 684 | 691 |
| 532.9666 | 1595.878 | 1595.8784 | 20 | WNDKPVALSIVQAR | 692 | 705 |
| 532.967 | 1595.8791 | 1595.8784 | 28 | WNDKPVALSIVQAR | 692 | 705 |
| 532.9672 | 1595.8797 | 1595.8784 | 15 | WNDKPVALSIVQAR | 692 | 705 |
| 798.9472 | 1595.8798 | 1595.8784 | 61 | WNDKPVALSIVQAR | 692 | 705 |
| 532.9673 | 1595.88 | 1595.8784 | 42 | WNDKPVALSIVQAR | 692 | 705 |

| PsaC | | | | | | |
| --- | --- | --- | --- | --- | --- | --- |
| Observed | Mr(expt) | Mr(calc) | Score | Peptide sequence | Amino acid position  start | Amino acid position  end |
| 823.3605 | 1644.7065 | 1644.7059 | 65 | IYDTCIGCTQCVR | 7 | 19 |
| 947.4117 | 1892.8088 | 1892.8107 | 61 | ACPTDVLEMVPWDGCK | 20 | 35 |
| 435.7416 | 869.4687 | 869.4719 | 83 | AGQIASAPR | 36 | 44 |
| 435.7427 | 869.4708 | 869.4719 | 26 | AGQIASAPR | 36 | 44 |
| 435.7429 | 869.4712 | 869.4719 | 31 | AGQIASAPR | 36 | 44 |
| 435.7434 | 869.4722 | 869.4719 | 56 | AGQIASAPR | 36 | 44 |
| 841.3926 | 1680.7706 | 1680.7712 | 25 | RCEAACPTDFLSVR | 53 | 66 |
| 763.3426 | 1524.6706 | 1524.6701 | 67 | CEAACPTDFLSVR | 54 | 66 |
| 513.267 | 1024.5194 | 1024.5189 | 40 | VYLGSETTR | 67 | 75 |

| PsaD | | | | | | |
| --- | --- | --- | --- | --- | --- | --- |
| Observed | Mr(expt) | Mr(calc) | Score | Peptide sequence | Amino acid position  start | Amino acid position  end |
| 764.4108 | 1526.8071 | 1526.8093 | 78 | AKTESAAPAVWTAPK | 36 | 50 |
| 509.9431 | 1526.8074 | 1526.8093 | 43 | AKTESAAPAVWTAPK | 36 | 50 |
| 509.9432 | 1526.8078 | 1526.8093 | 43 | AKTESAAPAVWTAPK | 36 | 50 |
| 664.8445 | 1327.6744 | 1327.6772 | 52 | TESAAPAVWTAPK | 38 | 50 |
| 664.8461 | 1327.6776 | 1327.6772 | 62 | TESAAPAVWTAPK | 38 | 50 |
| 664.8462 | 1327.6778 | 1327.6772 | 34 | TESAAPAVWTAPK | 38 | 50 |
| 1070.2273 | 3207.66 | 3207.6564 | 59 | TESAAPAVWTAPKLDPNTPSPIFGGSTGGLLR | 38 | 69 |
| 633.6685 | 1897.9837 | 1897.9898 | 28 | LDPNTPSPIFGGSTGGLLR | 51 | 69 |
| 633.6696 | 1897.987 | 1897.9898 | 41 | LDPNTPSPIFGGSTGGLLR | 51 | 69 |
| 633.6698 | 1897.9876 | 1897.9898 | 30 | LDPNTPSPIFGGSTGGLLR | 51 | 69 |
| 950.0012 | 1897.9879 | 1897.9898 | 70 | LDPNTPSPIFGGSTGGLLR | 51 | 69 |
| 633.67 | 1897.9883 | 1897.9898 | 57 | LDPNTPSPIFGGSTGGLLR | 51 | 69 |
| 633.6701 | 1897.9885 | 1897.9898 | 30 | LDPNTPSPIFGGSTGGLLR | 51 | 69 |
| 950.0015 | 1897.9885 | 1897.9898 | 88 | LDPNTPSPIFGGSTGGLLR | 51 | 69 |
| 633.6702 | 1897.9889 | 1897.9898 | 58 | LDPNTPSPIFGGSTGGLLR | 51 | 69 |
| 950.0018 | 1897.9891 | 1897.9898 | 70 | LDPNTPSPIFGGSTGGLLR | 51 | 69 |
| 633.6705 | 1897.9896 | 1897.9898 | 64 | LDPNTPSPIFGGSTGGLLR | 51 | 69 |
| 633.6705 | 1897.9896 | 1897.9898 | 25 | LDPNTPSPIFGGSTGGLLR | 51 | 69 |
| 950.0022 | 1897.9898 | 1897.9898 | 38 | LDPNTPSPIFGGSTGGLLR | 51 | 69 |
| 950.0024 | 1897.9902 | 1897.9898 | 64 | LDPNTPSPIFGGSTGGLLR | 51 | 69 |
| 950.0024 | 1897.9902 | 1897.9898 | 57 | LDPNTPSPIFGGSTGGLLR | 51 | 69 |
| 950.0024 | 1897.9903 | 1897.9898 | 102 | LDPNTPSPIFGGSTGGLLR | 51 | 69 |
| 950.0024 | 1897.9903 | 1897.9898 | 47 | LDPNTPSPIFGGSTGGLLR | 51 | 69 |
| 950.0026 | 1897.9906 | 1897.9898 | 67 | LDPNTPSPIFGGSTGGLLR | 51 | 69 |
| 950.0026 | 1897.9906 | 1897.9898 | 71 | LDPNTPSPIFGGSTGGLLR | 51 | 69 |
| 950.0027 | 1897.9908 | 1897.9898 | 55 | LDPNTPSPIFGGSTGGLLR | 51 | 69 |
| 950.0027 | 1897.9908 | 1897.9898 | 63 | LDPNTPSPIFGGSTGGLLR | 51 | 69 |
| 950.0028 | 1897.9911 | 1897.9898 | 50 | LDPNTPSPIFGGSTGGLLR | 51 | 69 |
| 950.0029 | 1897.9912 | 1897.9898 | 83 | LDPNTPSPIFGGSTGGLLR | 51 | 69 |
| 950.0029 | 1897.9912 | 1897.9898 | 43 | LDPNTPSPIFGGSTGGLLR | 51 | 69 |
| 950.0029 | 1897.9912 | 1897.9898 | 54 | LDPNTPSPIFGGSTGGLLR | 51 | 69 |
| 633.671 | 1897.9912 | 1897.9898 | 19 | LDPNTPSPIFGGSTGGLLR | 51 | 69 |
| 950.0029 | 1897.9913 | 1897.9898 | 79 | LDPNTPSPIFGGSTGGLLR | 51 | 69 |
| 950.003 | 1897.9915 | 1897.9898 | 65 | LDPNTPSPIFGGSTGGLLR | 51 | 69 |
| 950.0031 | 1897.9917 | 1897.9898 | 49 | LDPNTPSPIFGGSTGGLLR | 51 | 69 |
| 633.6712 | 1897.9918 | 1897.9898 | 19 | LDPNTPSPIFGGSTGGLLR | 51 | 69 |
| 950.0032 | 1897.9918 | 1897.9898 | 46 | LDPNTPSPIFGGSTGGLLR | 51 | 69 |
| 950.0032 | 1897.9918 | 1897.9898 | 52 | LDPNTPSPIFGGSTGGLLR | 51 | 69 |
| 950.0034 | 1897.9922 | 1897.9898 | 63 | LDPNTPSPIFGGSTGGLLR | 51 | 69 |
| 950.004 | 1897.9934 | 1897.9898 | 48 | LDPNTPSPIFGGSTGGLLR | 51 | 69 |
| 633.6719 | 1897.994 | 1897.9898 | 24 | LDPNTPSPIFGGSTGGLLR | 51 | 69 |
| 950.0046 | 1897.9947 | 1897.9898 | 65 | LDPNTPSPIFGGSTGGLLR | 51 | 69 |
| 950.0049 | 1897.9952 | 1897.9898 | 29 | LDPNTPSPIFGGSTGGLLR | 51 | 69 |
| 614.3199 | 1839.9378 | 1839.9407 | 16 | KAQVEEFYVLTWEAK | 70 | 84 |
| 920.9765 | 1839.9384 | 1839.9407 | 73 | KAQVEEFYVLTWEAK | 70 | 84 |
| 614.3202 | 1839.9389 | 1839.9407 | 20 | KAQVEEFYVLTWEAK | 70 | 84 |
| 614.3206 | 1839.94 | 1839.9407 | 23 | KAQVEEFYVLTWEAK | 70 | 84 |
| 920.9776 | 1839.9406 | 1839.9407 | 73 | KAQVEEFYVLTWEAK | 70 | 84 |
| 920.9779 | 1839.9413 | 1839.9407 | 63 | KAQVEEFYVLTWEAK | 70 | 84 |
| 920.978 | 1839.9414 | 1839.9407 | 116 | KAQVEEFYVLTWEAK | 70 | 84 |
| 614.3211 | 1839.9415 | 1839.9407 | 36 | KAQVEEFYVLTWEAK | 70 | 84 |
| 920.9782 | 1839.9417 | 1839.9407 | 117 | KAQVEEFYVLTWEAK | 70 | 84 |
| 614.3213 | 1839.942 | 1839.9407 | 36 | KAQVEEFYVLTWEAK | 70 | 84 |
| 920.9784 | 1839.9423 | 1839.9407 | 80 | KAQVEEFYVLTWEAK | 70 | 84 |
| 920.9796 | 1839.9447 | 1839.9407 | 72 | KAQVEEFYVLTWEAK | 70 | 84 |
| 920.9797 | 1839.9448 | 1839.9407 | 58 | KAQVEEFYVLTWEAK | 70 | 84 |
| 920.9797 | 1839.9448 | 1839.9407 | 76 | KAQVEEFYVLTWEAK | 70 | 84 |
| 920.9803 | 1839.946 | 1839.9407 | 67 | KAQVEEFYVLTWEAK | 70 | 84 |
| 920.9825 | 1839.9505 | 1839.9407 | 57 | KAQVEEFYVLTWEAK | 70 | 84 |
| 657.0204 | 1968.0393 | 1968.0356 | 66 | KAQVEEFYVLTWEAKK | 70 | 85 |
| 856.9255 | 1711.8365 | 1711.8457 | 42 | AQVEEFYVLTWEAK | 71 | 84 |
| 571.622 | 1711.844 | 1711.8457 | 61 | AQVEEFYVLTWEAK | 71 | 84 |
| 856.9294 | 1711.8442 | 1711.8457 | 72 | AQVEEFYVLTWEAK | 71 | 84 |
| 856.9295 | 1711.8444 | 1711.8457 | 85 | AQVEEFYVLTWEAK | 71 | 84 |
| 856.9296 | 1711.8447 | 1711.8457 | 74 | AQVEEFYVLTWEAK | 71 | 84 |
| 856.9301 | 1711.8457 | 1711.8457 | 88 | AQVEEFYVLTWEAK | 71 | 84 |
| 856.9302 | 1711.8458 | 1711.8457 | 72 | AQVEEFYVLTWEAK | 71 | 84 |
| 856.9304 | 1711.8462 | 1711.8457 | 69 | AQVEEFYVLTWEAK | 71 | 84 |
| 856.9304 | 1711.8463 | 1711.8457 | 79 | AQVEEFYVLTWEAK | 71 | 84 |
| 856.9306 | 1711.8466 | 1711.8457 | 79 | AQVEEFYVLTWEAK | 71 | 84 |
| 856.9307 | 1711.8469 | 1711.8457 | 99 | AQVEEFYVLTWEAK | 71 | 84 |
| 856.9308 | 1711.847 | 1711.8457 | 85 | AQVEEFYVLTWEAK | 71 | 84 |
| 856.9313 | 1711.848 | 1711.8457 | 72 | AQVEEFYVLTWEAK | 71 | 84 |
| 856.9321 | 1711.8497 | 1711.8457 | 25 | AQVEEFYVLTWEAK | 71 | 84 |
| 861.4387 | 1720.8629 | 1720.864 | 96 | KEAIFEMPTGGAAIMR | 85 | 100 |
| 574.6288 | 1720.8645 | 1720.864 | 49 | KEAIFEMPTGGAAIMR | 85 | 100 |
| 869.4374 | 1736.8603 | 1736.8589 | 98 | KEAIFEMPTGGAAIMR | 85 | 100 |
| 869.4377 | 1736.8609 | 1736.8589 | 77 | KEAIFEMPTGGAAIMR | 85 | 100 |
| 579.9609 | 1736.861 | 1736.8589 | 43 | KEAIFEMPTGGAAIMR | 85 | 100 |
| 579.9618 | 1736.8635 | 1736.8589 | 54 | KEAIFEMPTGGAAIMR | 85 | 100 |
| 585.2912 | 1752.8518 | 1752.8539 | 46 | KEAIFEMPTGGAAIMR | 85 | 100 |
| 585.292 | 1752.8543 | 1752.8539 | 16 | KEAIFEMPTGGAAIMR | 85 | 100 |
| 877.4347 | 1752.8548 | 1752.8539 | 84 | KEAIFEMPTGGAAIMR | 85 | 100 |
| 585.2927 | 1752.8562 | 1752.8539 | 55 | KEAIFEMPTGGAAIMR | 85 | 100 |
| 585.293 | 1752.8573 | 1752.8539 | 43 | KEAIFEMPTGGAAIMR | 85 | 100 |
| 627.9899 | 1880.9478 | 1880.9488 | 35 | KEAIFEMPTGGAAIMRK | 85 | 101 |
| 797.3906 | 1592.7667 | 1592.7691 | 67 | EAIFEMPTGGAAIMR | 86 | 100 |
| 797.3916 | 1592.7686 | 1592.7691 | 46 | EAIFEMPTGGAAIMR | 86 | 100 |
| 531.9308 | 1592.7707 | 1592.7691 | 40 | EAIFEMPTGGAAIMR | 86 | 100 |
| 797.3933 | 1592.7721 | 1592.7691 | 49 | EAIFEMPTGGAAIMR | 86 | 100 |
| 537.2616 | 1608.763 | 1608.764 | 37 | EAIFEMPTGGAAIMR | 86 | 100 |
| 805.3895 | 1608.7644 | 1608.764 | 45 | EAIFEMPTGGAAIMR | 86 | 100 |
| 805.3896 | 1608.7646 | 1608.764 | 62 | EAIFEMPTGGAAIMR | 86 | 100 |
| 805.39 | 1608.7653 | 1608.764 | 78 | EAIFEMPTGGAAIMR | 86 | 100 |
| 805.39 | 1608.7655 | 1608.764 | 32 | EAIFEMPTGGAAIMR | 86 | 100 |
| 805.39 | 1608.7655 | 1608.764 | 59 | EAIFEMPTGGAAIMR | 86 | 100 |
| 805.3907 | 1608.7668 | 1608.764 | 56 | EAIFEMPTGGAAIMR | 86 | 100 |
| 813.3871 | 1624.7596 | 1624.7589 | 66 | EAIFEMPTGGAAIMR | 86 | 100 |
| 813.3872 | 1624.7597 | 1624.7589 | 70 | EAIFEMPTGGAAIMR | 86 | 100 |
| 813.3873 | 1624.7601 | 1624.7589 | 52 | EAIFEMPTGGAAIMR | 86 | 100 |
| 542.5941 | 1624.7605 | 1624.7589 | 44 | EAIFEMPTGGAAIMR | 86 | 100 |
| 813.3881 | 1624.7617 | 1624.7589 | 60 | EAIFEMPTGGAAIMR | 86 | 100 |
| 813.3882 | 1624.7618 | 1624.7589 | 76 | EAIFEMPTGGAAIMR | 86 | 100 |
| 813.3886 | 1624.7627 | 1624.7589 | 55 | EAIFEMPTGGAAIMR | 86 | 100 |
| 385.2502 | 768.4859 | 768.4857 | 26 | KGPNLLK | 101 | 107 |
| 746.9009 | 1491.7873 | 1491.7868 | 75 | KEQCLALLNTFR | 111 | 122 |
| 498.2698 | 1491.7875 | 1491.7868 | 22 | KEQCLALLNTFR | 111 | 122 |
| 682.8528 | 1363.691 | 1363.6918 | 49 | EQCLALLNTFR | 112 | 122 |
| 391.1915 | 1170.5527 | 1170.5525 | 23 | MKLDGCIYR | 125 | 133 |
| 586.2841 | 1170.5537 | 1170.5525 | 46 | MKLDGCIYR | 125 | 133 |
| 448.7177 | 895.4209 | 895.4222 | 41 | LDGCIYR | 127 | 133 |
| 750.8931 | 1499.7716 | 1499.7773 | 61 | VFPSGEVQYLHPK | 134 | 146 |
| 750.8959 | 1499.7773 | 1499.7773 | 66 | VFPSGEVQYLHPK | 134 | 146 |
| 500.9334 | 1499.7784 | 1499.7773 | 32 | VFPSGEVQYLHPK | 134 | 146 |
| 750.8972 | 1499.7798 | 1499.7773 | 32 | VFPSGEVQYLHPK | 134 | 146 |
| 404.1973 | 806.38 | 806.381 | 26 | DGVYPEK | 147 | 153 |
| 383.5359 | 1147.5859 | 1147.5873 | 22 | DGVYPEKVNK | 147 | 156 |
| 453.2163 | 904.4181 | 904.4185 | 58 | VGANQNMR | 159 | 166 |

| PsaE | | | | | | |
| --- | --- | --- | --- | --- | --- | --- |
| Observed | Mr(expt) | Mr(calc) | Score | Peptide sequence | Amino acid position  start | Amino acid position  end |
| 591.9913 | 1772.952 | 1772.9533 | 41 | AEGEAAKPAAKPQVGPPR | 28 | 45 |
| 444.2456 | 1772.9533 | 1772.9533 | 23 | AEGEAAKPAAKPQVGPPR | 28 | 45 |
| 444.2456 | 1772.9535 | 1772.9533 | 19 | AEGEAAKPAAKPQVGPPR | 28 | 45 |
| 591.992 | 1772.9542 | 1772.9533 | 51 | AEGEAAKPAAKPQVGPPR | 28 | 45 |
| 887.485 | 1772.9555 | 1772.9533 | 66 | AEGEAAKPAAKPQVGPPR | 28 | 45 |
| 577.3094 | 2305.2085 | 2305.2212 | 24 | AEGEAAKPAAKPQVGPPRGTMVK | 28 | 50 |
| 596.6305 | 1786.8696 | 1786.8678 | 29 | ILRPESYWFNDYGK | 51 | 64 |
| 537.3048 | 1072.5949 | 1072.5877 | 52 | VISVDQTGVR | 65 | 74 |
| 366.7232 | 731.4319 | 731.433 | 21 | YPVVVR | 75 | 80 |
| 366.7237 | 731.4329 | 731.433 | 30 | YPVVVR | 75 | 80 |
| 366.7238 | 731.4331 | 731.433 | 25 | YPVVVR | 75 | 80 |
| 366.7258 | 731.437 | 731.433 | 27 | YPVVVR | 75 | 80 |
| 1010.9584 | 2019.9023 | 2019.9061 | 24 | VNYAGVSTNNYALDEVEY | 84 | 101 |
| 1010.9596 | 2019.9046 | 2019.9061 | 50 | VNYAGVSTNNYALDEVEY | 84 | 101 |
| 1010.9606 | 2019.9067 | 2019.9061 | 21 | VNYAGVSTNNYALDEVEY | 84 | 101 |
| 1010.9617 | 2019.9088 | 2019.9061 | 42 | VNYAGVSTNNYALDEVEY | 84 | 101 |
| 1010.9617 | 2019.9089 | 2019.9061 | 57 | VNYAGVSTNNYALDEVEY | 84 | 101 |
| 1010.9618 | 2019.909 | 2019.9061 | 95 | VNYAGVSTNNYALDEVEY | 84 | 101 |

| PsaF | | | | | | |
| --- | --- | --- | --- | --- | --- | --- |
| Observed | Mr(expt) | Mr(calc) | Score | Peptide sequence | Amino acid position  start | Amino acid position  end |
| 735.3961 | 2203.1665 | 2203.1558 | 53 | LKKYDPESAPALALQATMEK | 99 | 118 |
| 740.7231 | 2219.1476 | 2219.1507 | 64 | LKKYDPESAPALALQATMEK | 99 | 118 |
| 555.7952 | 2219.1516 | 2219.1507 | 25 | LKKYDPESAPALALQATMEK | 99 | 118 |
| 654.9994 | 1961.9765 | 1961.9768 | 56 | KYDPESAPALALQATMEK | 101 | 118 |
| 981.9982 | 1961.9818 | 1961.9768 | 86 | KYDPESAPALALQATMEK | 101 | 118 |
| 660.3296 | 1977.9669 | 1977.9717 | 34 | KYDPESAPALALQATMEK | 101 | 118 |
| 660.3304 | 1977.9695 | 1977.9717 | 54 | KYDPESAPALALQATMEK | 101 | 118 |
| 989.9922 | 1977.9698 | 1977.9717 | 61 | KYDPESAPALALQATMEK | 101 | 118 |
| 660.3309 | 1977.9708 | 1977.9717 | 41 | KYDPESAPALALQATMEK | 101 | 118 |
| 660.331 | 1977.9713 | 1977.9717 | 41 | KYDPESAPALALQATMEK | 101 | 118 |
| 989.9938 | 1977.9731 | 1977.9717 | 89 | KYDPESAPALALQATMEK | 101 | 118 |
| 660.332 | 1977.9741 | 1977.9717 | 49 | KYDPESAPALALQATMEK | 101 | 118 |
| 736.7128 | 2207.1165 | 2207.1143 | 40 | KYDPESAPALALQATMEKTK | 101 | 120 |
| 612.3009 | 1833.8809 | 1833.8818 | 56 | YDPESAPALALQATMEK | 102 | 118 |
| 917.9492 | 1833.8839 | 1833.8818 | 38 | YDPESAPALALQATMEK | 102 | 118 |
| 917.9493 | 1833.8841 | 1833.8818 | 99 | YDPESAPALALQATMEK | 102 | 118 |
| 917.9498 | 1833.885 | 1833.8818 | 57 | YDPESAPALALQATMEK | 102 | 118 |
| 925.945 | 1849.8753 | 1849.8767 | 28 | YDPESAPALALQATMEK | 102 | 118 |
| 925.9453 | 1849.8761 | 1849.8767 | 71 | YDPESAPALALQATMEK | 102 | 118 |
| 617.6334 | 1849.8784 | 1849.8767 | 28 | YDPESAPALALQATMEK | 102 | 118 |
| 925.9465 | 1849.8785 | 1849.8767 | 88 | YDPESAPALALQATMEK | 102 | 118 |
| 708.3348 | 1414.655 | 1414.6551 | 57 | FANYGESGLLCGK | 123 | 135 |
| 708.3354 | 1414.6562 | 1414.6551 | 72 | FANYGESGLLCGK | 123 | 135 |
| 441.2219 | 880.4293 | 880.429 | 24 | SYIQENK | 173 | 179 |
| 441.222 | 880.4295 | 880.429 | 30 | SYIQENK | 173 | 179 |
| 566.6485 | 1696.9237 | 1696.9247 | 43 | TASKPTEGEIIIDVPK | 180 | 195 |
| 849.4691 | 1696.9237 | 1696.9247 | 58 | TASKPTEGEIIIDVPK | 180 | 195 |
| 566.6487 | 1696.9242 | 1696.9247 | 52 | TASKPTEGEIIIDVPK | 180 | 195 |
| 566.6487 | 1696.9242 | 1696.9247 | 38 | TASKPTEGEIIIDVPK | 180 | 195 |
| 566.6492 | 1696.9257 | 1696.9247 | 48 | TASKPTEGEIIIDVPK | 180 | 195 |
| 849.4703 | 1696.926 | 1696.9247 | 62 | TASKPTEGEIIIDVPK | 180 | 195 |
| 849.4704 | 1696.9262 | 1696.9247 | 54 | TASKPTEGEIIIDVPK | 180 | 195 |
| 849.4706 | 1696.9267 | 1696.9247 | 74 | TASKPTEGEIIIDVPK | 180 | 195 |
| 566.6497 | 1696.9272 | 1696.9247 | 26 | TASKPTEGEIIIDVPK | 180 | 195 |
| 566.6504 | 1696.9295 | 1696.9247 | 18 | TASKPTEGEIIIDVPK | 180 | 195 |
| 1050.0806 | 2098.1466 | 2098.1649 | 22 | ALGLMFQAGAWPLLAGLELK | 196 | 215 |
| 836.4233 | 1670.8321 | 1670.8475 | 42 | NGTLTAPESEITVSPR | 216 | 231 |
| 557.9564 | 1670.8473 | 1670.8475 | 35 | NGTLTAPESEITVSPR | 216 | 231 |
| 836.4314 | 1670.8482 | 1670.8475 | 32 | NGTLTAPESEITVSPR | 216 | 231 |
| 557.9568 | 1670.8485 | 1670.8475 | 53 | NGTLTAPESEITVSPR | 216 | 231 |
| 836.4316 | 1670.8486 | 1670.8475 | 60 | NGTLTAPESEITVSPR | 216 | 231 |
| 836.4316 | 1670.8487 | 1670.8475 | 87 | NGTLTAPESEITVSPR | 216 | 231 |
| 836.4317 | 1670.8488 | 1670.8475 | 32 | NGTLTAPESEITVSPR | 216 | 231 |

| PsaG | | | | | | |
| --- | --- | --- | --- | --- | --- | --- |
| Observed | Mr(expt) | Mr(calc) | Score | Peptide sequence | Amino acid position  start | Amino acid position  end |
| 748.0427 | 2241.1062 | 2241.11 | 60 | AFDDVNVVMSASNALALYLGR | 36 | 56 |
| 1121.5631 | 2241.1117 | 2241.11 | 111 | AFDDVNVVMSASNALALYLGR | 36 | 56 |
| 535.2953 | 1068.576 | 1068.5757 | 29 | FVFLPYQR | 57 | 64 |
| 535.2955 | 1068.5765 | 1068.5757 | 31 | FVFLPYQR | 57 | 64 |
| 944.4409 | 1886.8672 | 1886.8694 | 87 | VGMPTQNGQTHFAAGDTR | 70 | 87 |
| 635.2956 | 1902.8649 | 1902.8643 | 37 | VGMPTQNGQTHFAAGDTR | 70 | 87 |
| 952.4407 | 1902.8668 | 1902.8643 | 83 | VGMPTQNGQTHFAAGDTR | 70 | 87 |

| PsaH | | | | | | |
| --- | --- | --- | --- | --- | --- | --- |
| Observed | Mr(expt) | Mr(calc) | Score | Peptide sequence | Amino acid position  start | Amino acid position  end |
| 1097.4791 | 3289.4156 | 3289.4212 | 31 | YGDESVYFDLSDVEATTGSWDVYGVDSASR | 72 | 101 |
| 977.4716 | 1952.9287 | 1952.938 | 39 | YPEQQAAFFEAAAQGLGR | 102 | 119 |
| 977.4749 | 1952.9353 | 1952.938 | 68 | YPEQQAAFFEAAAQGLGR | 102 | 119 |
| 651.9858 | 1952.9357 | 1952.938 | 40 | YPEQQAAFFEAAAQGLGR | 102 | 119 |
| 977.4753 | 1952.936 | 1952.938 | 49 | YPEQQAAFFEAAAQGLGR | 102 | 119 |
| 651.9867 | 1952.9382 | 1952.938 | 51 | YPEQQAAFFEAAAQGLGR | 102 | 119 |
| 651.9867 | 1952.9382 | 1952.938 | 24 | YPEQQAAFFEAAAQGLGR | 102 | 119 |
| 977.4769 | 1952.9393 | 1952.938 | 63 | YPEQQAAFFEAAAQGLGR | 102 | 119 |
| 977.477 | 1952.9394 | 1952.938 | 105 | YPEQQAAFFEAAAQGLGR | 102 | 119 |
| 977.4772 | 1952.9399 | 1952.938 | 94 | YPEQQAAFFEAAAQGLGR | 102 | 119 |
| 977.4774 | 1952.9403 | 1952.938 | 17 | YPEQQAAFFEAAAQGLGR | 102 | 119 |
| 977.4777 | 1952.9408 | 1952.938 | 20 | YPEQQAAFFEAAAQGLGR | 102 | 119 |
| 977.478 | 1952.9414 | 1952.938 | 76 | YPEQQAAFFEAAAQGLGR | 102 | 119 |
| 977.4781 | 1952.9416 | 1952.938 | 80 | YPEQQAAFFEAAAQGLGR | 102 | 119 |
| 977.4784 | 1952.9422 | 1952.938 | 83 | YPEQQAAFFEAAAQGLGR | 102 | 119 |
| 977.4786 | 1952.9427 | 1952.938 | 39 | YPEQQAAFFEAAAQGLGR | 102 | 119 |
| 704.0194 | 2109.0364 | 2109.0392 | 21 | YPEQQAAFFEAAAQGLGRR | 102 | 120 |
| 756.0983 | 2265.273 | 2265.2733 | 87 | REAVYSVLAVSAGLLTVAYGVK | 120 | 141 |
| 704.0645 | 2109.1717 | 2109.1722 | 56 | EAVYSVLAVSAGLLTVAYGVK | 121 | 141 |
| 1055.5938 | 2109.1729 | 2109.1722 | 58 | EAVYSVLAVSAGLLTVAYGVK | 121 | 141 |
| 789.4495 | 2365.3266 | 2365.3257 | 30 | EAVYSVLAVSAGLLTVAYGVKGAK | 121 | 144 |
| 986.5546 | 1971.0947 | 1971.0902 | 75 | DAKLPITVGPQQPAQVGPR | 145 | 163 |
| 658.0391 | 1971.0954 | 1971.0902 | 59 | DAKLPITVGPQQPAQVGPR | 145 | 163 |
| 829.4694 | 1656.9242 | 1656.9312 | 61 | LPITVGPQQPAQVGPR | 148 | 163 |
| 829.47 | 1656.9254 | 1656.9312 | 64 | LPITVGPQQPAQVGPR | 148 | 163 |
| 829.4714 | 1656.9283 | 1656.9312 | 19 | LPITVGPQQPAQVGPR | 148 | 163 |
| 829.4717 | 1656.9288 | 1656.9312 | 52 | LPITVGPQQPAQVGPR | 148 | 163 |
| 829.473 | 1656.9314 | 1656.9312 | 54 | LPITVGPQQPAQVGPR | 148 | 163 |
| 829.4731 | 1656.9316 | 1656.9312 | 23 | LPITVGPQQPAQVGPR | 148 | 163 |
| 829.4734 | 1656.9322 | 1656.9312 | 52 | LPITVGPQQPAQVGPR | 148 | 163 |
| 829.4734 | 1656.9323 | 1656.9312 | 42 | LPITVGPQQPAQVGPR | 148 | 163 |
| 829.4736 | 1656.9327 | 1656.9312 | 51 | LPITVGPQQPAQVGPR | 148 | 163 |
| 829.474 | 1656.9334 | 1656.9312 | 59 | LPITVGPQQPAQVGPR | 148 | 163 |
| 829.474 | 1656.9334 | 1656.9312 | 56 | LPITVGPQQPAQVGPR | 148 | 163 |
| 829.4742 | 1656.9338 | 1656.9312 | 63 | LPITVGPQQPAQVGPR | 148 | 163 |
| 829.4743 | 1656.934 | 1656.9312 | 41 | LPITVGPQQPAQVGPR | 148 | 163 |
| 829.4746 | 1656.9345 | 1656.9312 | 73 | LPITVGPQQPAQVGPR | 148 | 163 |
| 829.4746 | 1656.9345 | 1656.9312 | 63 | LPITVGPQQPAQVGPR | 148 | 163 |
| 829.4747 | 1656.9348 | 1656.9312 | 86 | LPITVGPQQPAQVGPR | 148 | 163 |
| 829.4749 | 1656.9353 | 1656.9312 | 76 | LPITVGPQQPAQVGPR | 148 | 163 |
| 829.475 | 1656.9355 | 1656.9312 | 45 | LPITVGPQQPAQVGPR | 148 | 163 |
| 553.3192 | 1656.9358 | 1656.9312 | 57 | LPITVGPQQPAQVGPR | 148 | 163 |

| PsaJ | | | | | | |
| --- | --- | --- | --- | --- | --- | --- |
| Observed | Mr(expt) | Mr(calc) | Score | Peptide sequence | Amino acid position  start | Amino acid position  end |
| 627.8234 | 1253.6322 | 1253.6332 | 54 | FFPDALSFPLT | 32 | 42 |
| 627.8243 | 1253.634 | 1253.6332 | 34 | FFPDALSFPLT | 32 | 42 |

| PsaK | | | | | | |
| --- | --- | --- | --- | --- | --- | --- |
| Observed | Mr(expt) | Mr(calc) | Score | Peptide sequence | Amino acid position  start | Amino acid position  end |
| 439.7444 | 877.4743 | 877.4732 | 42 | FGLMPSVK | 68 | 75 |
| 447.7401 | 893.4657 | 893.4681 | 29 | FGLMPSVK | 68 | 75 |
| 447.7413 | 893.468 | 893.4681 | 30 | FGLMPSVK | 68 | 75 |
| 447.7414 | 893.4682 | 893.4681 | 34 | FGLMPSVK | 68 | 75 |
| 447.7417 | 893.4688 | 893.4681 | 17 | FGLMPSVK | 68 | 75 |
| 447.7417 | 893.4688 | 893.4681 | 34 | FGLMPSVK | 68 | 75 |
| 668.9893 | 2003.946 | 2003.9483 | 19 | KNFNGASSHTAMVEQANAK | 76 | 94 |
| 668.9915 | 2003.9527 | 2003.9483 | 45 | KNFNGASSHTAMVEQANAK | 76 | 94 |
| 505.9932 | 2019.9438 | 2019.9432 | 25 | KNFNGASSHTAMVEQANAK | 76 | 94 |
| 674.3226 | 2019.9459 | 2019.9432 | 55 | KNFNGASSHTAMVEQANAK | 76 | 94 |
| 938.9314 | 1875.8482 | 1875.8533 | 81 | NFNGASSHTAMVEQANAK | 77 | 94 |
| 626.2913 | 1875.8521 | 1875.8533 | 65 | NFNGASSHTAMVEQANAK | 77 | 94 |
| 631.6226 | 1891.846 | 1891.8483 | 52 | NFNGASSHTAMVEQANAK | 77 | 94 |
| 946.9308 | 1891.847 | 1891.8483 | 89 | NFNGASSHTAMVEQANAK | 77 | 94 |

| PsaL | | | | | | |
| --- | --- | --- | --- | --- | --- | --- |
| Observed | Mr(expt) | Mr(calc) | Score | Peptide sequence | Amino acid position  start | Amino acid position  end |
| 1000.7762 | 3999.0756 | 3999.0816 | 106 | VQIVKPINGDPFIGMLETPVTSSPDVANFLSNLPAYR | 49 | 85 |
| 1334.0333 | 3999.0782 | 3999.0816 | 46 | VQIVKPINGDPFIGMLETPVTSSPDVANFLSNLPAYR | 49 | 85 |
| 1000.7778 | 3999.0822 | 3999.0816 | 60 | VQIVKPINGDPFIGMLETPVTSSPDVANFLSNLPAYR | 49 | 85 |
| 1004.7764 | 4015.0766 | 4015.0765 | 77 | VQIVKPINGDPFIGMLETPVTSSPDVANFLSNLPAYR | 49 | 85 |
| 1339.3668 | 4015.0786 | 4015.0765 | 74 | VQIVKPINGDPFIGMLETPVTSSPDVANFLSNLPAYR | 49 | 85 |
| 1339.3677 | 4015.0812 | 4015.0765 | 82 | VQIVKPINGDPFIGMLETPVTSSPDVANFLSNLPAYR | 49 | 85 |
| 1339.3688 | 4015.0845 | 4015.0765 | 87 | VQIVKPINGDPFIGMLETPVTSSPDVANFLSNLPAYR | 49 | 85 |
| 413.7604 | 825.5061 | 825.5072 | 34 | TGVAPLLR | 86 | 93 |
| 413.7606 | 825.5067 | 825.5072 | 39 | TGVAPLLR | 86 | 93 |
| 413.761 | 825.5074 | 825.5072 | 24 | TGVAPLLR | 86 | 93 |
| 413.7611 | 825.5076 | 825.5072 | 28 | TGVAPLLR | 86 | 93 |
| 413.7612 | 825.5078 | 825.5072 | 36 | TGVAPLLR | 86 | 93 |
| 671.3859 | 2681.5146 | 2681.5058 | 18 | TGVAPLLRGVEVGLAHGFFVTGPFIK | 86 | 111 |
| 625.6755 | 1874.0048 | 1874.0091 | 31 | GVEVGLAHGFFVTGPFIK | 94 | 111 |
| 625.6756 | 1874.005 | 1874.0091 | 21 | GVEVGLAHGFFVTGPFIK | 94 | 111 |
| 625.6771 | 1874.0094 | 1874.0091 | 50 | GVEVGLAHGFFVTGPFIK | 94 | 111 |
| 625.6772 | 1874.0099 | 1874.0091 | 37 | GVEVGLAHGFFVTGPFIK | 94 | 111 |
| 938.0126 | 1874.0107 | 1874.0091 | 36 | GVEVGLAHGFFVTGPFIK | 94 | 111 |
| 938.0128 | 1874.0111 | 1874.0091 | 36 | GVEVGLAHGFFVTGPFIK | 94 | 111 |
| 625.6778 | 1874.0116 | 1874.0091 | 25 | GVEVGLAHGFFVTGPFIK | 94 | 111 |
| 625.6779 | 1874.0118 | 1874.0091 | 40 | GVEVGLAHGFFVTGPFIK | 94 | 111 |
| 938.0133 | 1874.0121 | 1874.0091 | 79 | GVEVGLAHGFFVTGPFIK | 94 | 111 |
| 625.678 | 1874.0123 | 1874.0091 | 34 | GVEVGLAHGFFVTGPFIK | 94 | 111 |
| 625.678 | 1874.0123 | 1874.0091 | 31 | GVEVGLAHGFFVTGPFIK | 94 | 111 |
| 938.0147 | 1874.0149 | 1874.0091 | 41 | GVEVGLAHGFFVTGPFIK | 94 | 111 |
| 603.5945 | 2410.3488 | 2410.3526 | 19 | GVEVGLAHGFFVTGPFIKLGPLR | 94 | 116 |
| 804.4575 | 2410.3507 | 2410.3526 | 50 | GVEVGLAHGFFVTGPFIKLGPLR | 94 | 116 |
| 387.2235 | 772.4324 | 772.4331 | 23 | EEVVGLK | 151 | 157 |
| 387.2244 | 772.4343 | 772.4331 | 43 | EEVVGLK | 151 | 157 |

| PsaN | | | | | | |
| --- | --- | --- | --- | --- | --- | --- |
| Observed | Mr(expt) | Mr(calc) | Score | Peptide sequence | Amino acid position  start | Amino acid position  end |
| 605.3005 | 1208.5864 | 1208.5898 | 54 | RAATSSANFER | 68 | 78 |
| 403.8703 | 1208.5892 | 1208.5898 | 29 | RAATSSANFER | 68 | 78 |
| 527.2506 | 1052.4865 | 1052.4887 | 59 | AATSSANFER | 69 | 78 |
| 527.2515 | 1052.4884 | 1052.4887 | 53 | AATSSANFER | 69 | 78 |
| 527.2518 | 1052.4891 | 1052.4887 | 79 | AATSSANFER | 69 | 78 |
| 848.7246 | 2543.1518 | 2543.1573 | 47 | LVTDGFCQFPQNIFGCQNAAEK | 81 | 102 |
| 1272.5857 | 2543.1568 | 2543.1573 | 57 | LVTDGFCQFPQNIFGCQNAAEK | 81 | 102 |
| 848.7264 | 2543.1573 | 2543.1573 | 39 | LVTDGFCQFPQNIFGCQNAAEK | 81 | 102 |
| 848.7264 | 2543.1575 | 2543.1573 | 28 | LVTDGFCQFPQNIFGCQNAAEK | 81 | 102 |
| 848.7267 | 2543.1582 | 2543.1573 | 60 | LVTDGFCQFPQNIFGCQNAAEK | 81 | 102 |
| 1272.5869 | 2543.1593 | 2543.1573 | 60 | LVTDGFCQFPQNIFGCQNAAEK | 81 | 102 |
| 1272.5872 | 2543.1598 | 2543.1573 | 50 | LVTDGFCQFPQNIFGCQNAAEK | 81 | 102 |
| 848.7273 | 2543.16 | 2543.1573 | 44 | LVTDGFCQFPQNIFGCQNAAEK | 81 | 102 |
| 1272.5874 | 2543.1602 | 2543.1573 | 58 | LVTDGFCQFPQNIFGCQNAAEK | 81 | 102 |
| 848.7277 | 2543.1612 | 2543.1573 | 72 | LVTDGFCQFPQNIFGCQNAAEK | 81 | 102 |
| 848.7277 | 2543.1612 | 2543.1573 | 19 | LVTDGFCQFPQNIFGCQNAAEK | 81 | 102 |
| 848.7278 | 2543.1617 | 2543.1573 | 33 | LVTDGFCQFPQNIFGCQNAAEK | 81 | 102 |
| 1272.5883 | 2543.162 | 2543.1573 | 42 | LVTDGFCQFPQNIFGCQNAAEK | 81 | 102 |
| 1272.5886 | 2543.1627 | 2543.1573 | 49 | LVTDGFCQFPQNIFGCQNAAEK | 81 | 102 |
| 848.7292 | 2543.1659 | 2543.1573 | 34 | LVTDGFCQFPQNIFGCQNAAEK | 81 | 102 |
| 929.8967 | 1857.7789 | 1857.7761 | 104 | FLSDDMAIECEGTADGK | 107 | 123 |
| 937.892 | 1873.7694 | 1873.771 | 109 | FLSDDMAIECEGTADGK | 107 | 123 |
| 937.8922 | 1873.7699 | 1873.771 | 116 | FLSDDMAIECEGTADGK | 107 | 123 |
| 625.5976 | 1873.771 | 1873.771 | 47 | FLSDDMAIECEGTADGK | 107 | 123 |
| 937.8934 | 1873.7722 | 1873.771 | 27 | FLSDDMAIECEGTADGK | 107 | 123 |
| 937.8934 | 1873.7723 | 1873.771 | 94 | FLSDDMAIECEGTADGK | 107 | 123 |
| 937.8935 | 1873.7724 | 1873.771 | 120 | FLSDDMAIECEGTADGK | 107 | 123 |
| 937.8939 | 1873.7733 | 1873.771 | 100 | FLSDDMAIECEGTADGK | 107 | 123 |
| 937.8943 | 1873.774 | 1873.771 | 100 | FLSDDMAIECEGTADGK | 107 | 123 |
| 937.8952 | 1873.7758 | 1873.771 | 110 | FLSDDMAIECEGTADGK | 107 | 123 |
| 813.3451 | 2437.0134 | 2437.0083 | 30 | FLSDDMAIECEGTADGKTCSSK | 107 | 128 |
| 546.7888 | 1091.5631 | 1091.5651 | 18 | APGAYPSFLGL | 129 | 139 |
| 546.7891 | 1091.5636 | 1091.5651 | 35 | APGAYPSFLGL | 129 | 139 |
| 546.7897 | 1091.5648 | 1091.5651 | 51 | APGAYPSFLGL | 129 | 139 |
| 546.7897 | 1091.5648 | 1091.5651 | 38 | APGAYPSFLGL | 129 | 139 |
| 546.7898 | 1091.565 | 1091.5651 | 50 | APGAYPSFLGL | 129 | 139 |
| 546.7898 | 1091.565 | 1091.5651 | 53 | APGAYPSFLGL | 129 | 139 |
| 546.79 | 1091.5655 | 1091.5651 | 41 | APGAYPSFLGL | 129 | 139 |
| 546.7901 | 1091.5656 | 1091.5651 | 58 | APGAYPSFLGL | 129 | 139 |
| 546.7906 | 1091.5667 | 1091.5651 | 24 | APGAYPSFLGL | 129 | 139 |

| PsaO | | | | | | |
| --- | --- | --- | --- | --- | --- | --- |
| Observed | Mr(expt) | Mr(calc) | Score | Peptide sequence | Amino acid position  start | Amino acid position  end |
| 413.1749 | 824.3352 | 824.3341 | 18 | FDDDWK | 43 | 48 |
| 477.2203 | 952.4261 | 952.429 | 36 | FDDDWKK | 43 | 49 |
| 407.198 | 812.3814 | 812.3817 | 45 | KQGYFGN | 130 | 136 |

| Lhca1 | | | | | | |
| --- | --- | --- | --- | --- | --- | --- |
| Observed | Mr(expt) | Mr(calc) | Score | Peptide sequence | Amino acid position  start | Amino acid position  end |
| 496.0011 | 1979.9753 | 1979.9788 | 41 | ASARPMWFPGAEAPAHLR | 28 | 45 |
| 661.0003 | 1979.9791 | 1979.9788 | 35 | ASARPMWFPGAEAPAHLR | 28 | 45 |
| 859.4075 | 2575.2006 | 2575.2013 | 25 | GDLPCDYGFDPLNLGEKPDNLAR | 46 | 68 |
| 1288.6096 | 2575.2047 | 2575.2013 | 32 | GDLPCDYGFDPLNLGEKPDNLAR | 46 | 68 |
| 859.4089 | 2575.205 | 2575.2013 | 39 | GDLPCDYGFDPLNLGEKPDNLAR | 46 | 68 |
| 1288.6101 | 2575.2057 | 2575.2013 | 38 | GDLPCDYGFDPLNLGEKPDNLAR | 46 | 68 |
| 425.8803 | 1274.6191 | 1274.6189 | 31 | YREAELMHAR | 69 | 78 |
| 638.3172 | 1274.6198 | 1274.6189 | 23 | YREAELMHAR | 69 | 78 |
| 646.313 | 1290.6114 | 1290.6139 | 32 | YREAELMHAR | 69 | 78 |
| 431.2121 | 1290.6146 | 1290.6139 | 22 | YREAELMHAR | 69 | 78 |
| 478.7327 | 955.4508 | 955.4545 | 42 | EAELMHAR | 71 | 78 |
| 478.7353 | 955.4561 | 955.4545 | 32 | EAELMHAR | 71 | 78 |
| 838.1448 | 3348.55 | 3348.5544 | 34 | WAMMGVAGAVGVEIAGQGDWASAQPTTWDATAK | 79 | 111 |
| 1122.5232 | 3364.5477 | 3364.5493 | 72 | WAMMGVAGAVGVEIAGQGDWASAQPTTWDATAK | 79 | 111 |
| 842.1446 | 3364.5495 | 3364.5493 | 48 | WAMMGVAGAVGVEIAGQGDWASAQPTTWDATAK | 79 | 111 |
| 991.5258 | 2971.5556 | 2971.5556 | 83 | YLGNETHAPLFAVIGVNGVLVAFAESQR | 112 | 139 |
| 743.897 | 2971.5588 | 2971.5556 | 70 | YLGNETHAPLFAVIGVNGVLVAFAESQR | 112 | 139 |
| 704.8568 | 1407.6991 | 1407.7034 | 22 | LYPGGSFDPAGLSK | 148 | 161 |
| 704.8572 | 1407.6998 | 1407.7034 | 35 | LYPGGSFDPAGLSK | 148 | 161 |
| 704.8575 | 1407.7004 | 1407.7034 | 26 | LYPGGSFDPAGLSK | 148 | 161 |
| 704.859 | 1407.7033 | 1407.7034 | 39 | LYPGGSFDPAGLSK | 148 | 161 |
| 704.859 | 1407.7035 | 1407.7034 | 29 | LYPGGSFDPAGLSK | 148 | 161 |
| 704.8591 | 1407.7036 | 1407.7034 | 21 | LYPGGSFDPAGLSK | 148 | 161 |
| 704.8591 | 1407.7036 | 1407.7034 | 23 | LYPGGSFDPAGLSK | 148 | 161 |
| 704.8591 | 1407.7037 | 1407.7034 | 41 | LYPGGSFDPAGLSK | 148 | 161 |
| 704.8592 | 1407.7039 | 1407.7034 | 32 | LYPGGSFDPAGLSK | 148 | 161 |
| 704.8595 | 1407.7044 | 1407.7034 | 62 | LYPGGSFDPAGLSK | 148 | 161 |
| 704.8596 | 1407.7047 | 1407.7034 | 55 | LYPGGSFDPAGLSK | 148 | 161 |
| 704.8596 | 1407.7047 | 1407.7034 | 29 | LYPGGSFDPAGLSK | 148 | 161 |
| 704.8596 | 1407.7047 | 1407.7034 | 31 | LYPGGSFDPAGLSK | 148 | 161 |
| 704.8597 | 1407.7049 | 1407.7034 | 59 | LYPGGSFDPAGLSK | 148 | 161 |
| 704.8602 | 1407.7059 | 1407.7034 | 36 | LYPGGSFDPAGLSK | 148 | 161 |
| 469.2526 | 936.4906 | 936.4916 | 36 | GKDFETLK | 162 | 169 |
| 469.2533 | 936.4921 | 936.4916 | 22 | GKDFETLK | 162 | 169 |
| 365.2042 | 1092.5907 | 1092.5927 | 19 | GKDFETLKR | 162 | 170 |
| 365.2047 | 1092.5922 | 1092.5927 | 30 | GKDFETLKR | 162 | 170 |
| 547.3041 | 1092.5936 | 1092.5927 | 46 | GKDFETLKR | 162 | 170 |
| 376.6945 | 751.3744 | 751.3752 | 33 | DFETLK | 164 | 169 |
| 454.7437 | 907.4729 | 907.4763 | 24 | DFETLKR | 164 | 170 |
| 829.7373 | 2486.1901 | 2486.1909 | 69 | VAMMAFFGIMAQHQADPSGPGPVK | 178 | 201 |
| 622.5549 | 2486.1906 | 2486.1909 | 40 | VAMMAFFGIMAQHQADPSGPGPVK | 178 | 201 |
| 835.069 | 2502.1853 | 2502.1858 | 33 | VAMMAFFGIMAQHQADPSGPGPVK | 178 | 201 |
| 626.5541 | 2502.1872 | 2502.1858 | 59 | VAMMAFFGIMAQHQADPSGPGPVK | 178 | 201 |
| 835.0697 | 2502.1873 | 2502.1858 | 52 | VAMMAFFGIMAQHQADPSGPGPVK | 178 | 201 |
| 835.0704 | 2502.1893 | 2502.1858 | 45 | VAMMAFFGIMAQHQADPSGPGPVK | 178 | 201 |
| 840.4009 | 2518.1808 | 2518.1807 | 52 | VAMMAFFGIMAQHQADPSGPGPVK | 178 | 201 |
| 634.5516 | 2534.1775 | 2534.1756 | 42 | VAMMAFFGIMAQHQADPSGPGPVK | 178 | 201 |
| 845.7333 | 2534.1782 | 2534.1756 | 18 | VAMMAFFGIMAQHQADPSGPGPVK | 178 | 201 |
| 845.7338 | 2534.1795 | 2534.1756 | 31 | VAMMAFFGIMAQHQADPSGPGPVK | 178 | 201 |
| 845.735 | 2534.1831 | 2534.1756 | 28 | VAMMAFFGIMAQHQADPSGPGPVK | 178 | 201 |
| 918.4589 | 2752.3548 | 2752.3544 | 26 | QLANHLADPWHVNVCTNPSAIPWL | 202 | 225 |

| Lhca2 | | | | | | |
| --- | --- | --- | --- | --- | --- | --- |
| Observed | Mr(expt) | Mr(calc) | Score | Peptide sequence | Amino acid position  start | Amino acid position  end |
| 728.0994 | 2908.3686 | 2908.3702 | 27 | APAPHLDGSLPGDFGFDPLSLSADPEMR | 44 | 71 |
| 970.4646 | 2908.372 | 2908.3702 | 60 | APAPHLDGSLPGDFGFDPLSLSADPEMR | 44 | 71 |
| 732.0977 | 2924.3618 | 2924.3651 | 39 | APAPHLDGSLPGDFGFDPLSLSADPEMR | 44 | 71 |
| 975.7947 | 2924.3622 | 2924.3651 | 39 | APAPHLDGSLPGDFGFDPLSLSADPEMR | 44 | 71 |
| 975.7953 | 2924.364 | 2924.3651 | 31 | APAPHLDGSLPGDFGFDPLSLSADPEMR | 44 | 71 |
| 975.7963 | 2924.3672 | 2924.3651 | 53 | APAPHLDGSLPGDFGFDPLSLSADPEMR | 44 | 71 |
| 732.0996 | 2924.3691 | 2924.3651 | 53 | APAPHLDGSLPGDFGFDPLSLSADPEMR | 44 | 71 |
| 975.7972 | 2924.3697 | 2924.3651 | 52 | APAPHLDGSLPGDFGFDPLSLSADPEMR | 44 | 71 |
| 975.799 | 2924.3752 | 2924.3651 | 31 | APAPHLDGSLPGDFGFDPLSLSADPEMR | 44 | 71 |
| 764.1213 | 3052.4563 | 3052.46 | 23 | APAPHLDGSLPGDFGFDPLSLSADPEMRK | 44 | 72 |
| 1018.4956 | 3052.465 | 3052.46 | 56 | APAPHLDGSLPGDFGFDPLSLSADPEMRK | 44 | 72 |
| 764.1239 | 3052.4665 | 3052.46 | 22 | APAPHLDGSLPGDFGFDPLSLSADPEMRK | 44 | 72 |
| 374.9497 | 1495.7696 | 1495.7718 | 40 | KWMVQAELQHAR | 72 | 83 |
| 499.5978 | 1495.7715 | 1495.7718 | 63 | KWMVQAELQHAR | 72 | 83 |
| 378.9483 | 1511.7642 | 1511.7667 | 29 | KWMVQAELQHAR | 72 | 83 |
| 504.9287 | 1511.7643 | 1511.7667 | 28 | KWMVQAELQHAR | 72 | 83 |
| 756.8905 | 1511.7664 | 1511.7667 | 61 | KWMVQAELQHAR | 72 | 83 |
| 504.9296 | 1511.7669 | 1511.7667 | 57 | KWMVQAELQHAR | 72 | 83 |
| 504.9308 | 1511.7707 | 1511.7667 | 37 | KWMVQAELQHAR | 72 | 83 |
| 456.8998 | 1367.6777 | 1367.6768 | 30 | WMVQAELQHAR | 73 | 83 |
| 684.8472 | 1367.6798 | 1367.6768 | 63 | WMVQAELQHAR | 73 | 83 |
| 462.2298 | 1383.6676 | 1383.6717 | 45 | WMVQAELQHAR | 73 | 83 |
| 462.2302 | 1383.6687 | 1383.6717 | 31 | WMVQAELQHAR | 73 | 83 |
| 462.231 | 1383.6711 | 1383.6717 | 37 | WMVQAELQHAR | 73 | 83 |
| 692.8432 | 1383.6718 | 1383.6717 | 54 | WMVQAELQHAR | 73 | 83 |
| 863.98 | 1725.9454 | 1725.9487 | 73 | WAMLGVAGAVAPELLTK | 84 | 100 |
| 863.9801 | 1725.9456 | 1725.9487 | 92 | WAMLGVAGAVAPELLTK | 84 | 100 |
| 576.3229 | 1725.9468 | 1725.9487 | 73 | WAMLGVAGAVAPELLTK | 84 | 100 |
| 863.9814 | 1725.9483 | 1725.9487 | 74 | WAMLGVAGAVAPELLTK | 84 | 100 |
| 863.9816 | 1725.9487 | 1725.9487 | 47 | WAMLGVAGAVAPELLTK | 84 | 100 |
| 863.9816 | 1725.9487 | 1725.9487 | 49 | WAMLGVAGAVAPELLTK | 84 | 100 |
| 863.9818 | 1725.9489 | 1725.9487 | 80 | WAMLGVAGAVAPELLTK | 84 | 100 |
| 863.9819 | 1725.9493 | 1725.9487 | 86 | WAMLGVAGAVAPELLTK | 84 | 100 |
| 863.9821 | 1725.9496 | 1725.9487 | 83 | WAMLGVAGAVAPELLTK | 84 | 100 |
| 863.9821 | 1725.9497 | 1725.9487 | 69 | WAMLGVAGAVAPELLTK | 84 | 100 |
| 863.9824 | 1725.9502 | 1725.9487 | 84 | WAMLGVAGAVAPELLTK | 84 | 100 |
| 863.9824 | 1725.9502 | 1725.9487 | 68 | WAMLGVAGAVAPELLTK | 84 | 100 |
| 863.9824 | 1725.9503 | 1725.9487 | 56 | WAMLGVAGAVAPELLTK | 84 | 100 |
| 863.9826 | 1725.9506 | 1725.9487 | 64 | WAMLGVAGAVAPELLTK | 84 | 100 |
| 863.9827 | 1725.9508 | 1725.9487 | 58 | WAMLGVAGAVAPELLTK | 84 | 100 |
| 863.9827 | 1725.9509 | 1725.9487 | 71 | WAMLGVAGAVAPELLTK | 84 | 100 |
| 863.9828 | 1725.951 | 1725.9487 | 63 | WAMLGVAGAVAPELLTK | 84 | 100 |
| 576.3248 | 1725.9525 | 1725.9487 | 43 | WAMLGVAGAVAPELLTK | 84 | 100 |
| 863.9836 | 1725.9527 | 1725.9487 | 78 | WAMLGVAGAVAPELLTK | 84 | 100 |
| 871.9784 | 1741.9422 | 1741.9437 | 41 | WAMLGVAGAVAPELLTK | 84 | 100 |
| 871.9785 | 1741.9425 | 1741.9437 | 41 | WAMLGVAGAVAPELLTK | 84 | 100 |
| 581.655 | 1741.9431 | 1741.9437 | 45 | WAMLGVAGAVAPELLTK | 84 | 100 |
| 871.9789 | 1741.9432 | 1741.9437 | 38 | WAMLGVAGAVAPELLTK | 84 | 100 |
| 581.6552 | 1741.9436 | 1741.9437 | 36 | WAMLGVAGAVAPELLTK | 84 | 100 |
| 871.9791 | 1741.9437 | 1741.9437 | 50 | WAMLGVAGAVAPELLTK | 84 | 100 |
| 871.9793 | 1741.9441 | 1741.9437 | 56 | WAMLGVAGAVAPELLTK | 84 | 100 |
| 871.9794 | 1741.9443 | 1741.9437 | 47 | WAMLGVAGAVAPELLTK | 84 | 100 |
| 871.9794 | 1741.9443 | 1741.9437 | 65 | WAMLGVAGAVAPELLTK | 84 | 100 |
| 871.9795 | 1741.9444 | 1741.9437 | 70 | WAMLGVAGAVAPELLTK | 84 | 100 |
| 871.9795 | 1741.9444 | 1741.9437 | 59 | WAMLGVAGAVAPELLTK | 84 | 100 |
| 871.9797 | 1741.9449 | 1741.9437 | 62 | WAMLGVAGAVAPELLTK | 84 | 100 |
| 871.9798 | 1741.945 | 1741.9437 | 69 | WAMLGVAGAVAPELLTK | 84 | 100 |
| 871.9799 | 1741.9453 | 1741.9437 | 89 | WAMLGVAGAVAPELLTK | 84 | 100 |
| 581.6558 | 1741.9455 | 1741.9437 | 29 | WAMLGVAGAVAPELLTK | 84 | 100 |
| 871.9801 | 1741.9456 | 1741.9437 | 56 | WAMLGVAGAVAPELLTK | 84 | 100 |
| 581.6559 | 1741.9458 | 1741.9437 | 27 | WAMLGVAGAVAPELLTK | 84 | 100 |
| 871.9802 | 1741.9459 | 1741.9437 | 53 | WAMLGVAGAVAPELLTK | 84 | 100 |
| 871.9806 | 1741.9467 | 1741.9437 | 44 | WAMLGVAGAVAPELLTK | 84 | 100 |
| 871.9811 | 1741.9476 | 1741.9437 | 44 | WAMLGVAGAVAPELLTK | 84 | 100 |
| 871.9811 | 1741.9477 | 1741.9437 | 57 | WAMLGVAGAVAPELLTK | 84 | 100 |
| 871.9813 | 1741.948 | 1741.9437 | 47 | WAMLGVAGAVAPELLTK | 84 | 100 |
| 1007.6903 | 4026.7321 | 4026.7313 | 73 | WQDMKNPGSMNTDPLFGYNSNDTNTDVGYPGGLFDK | 140 | 175 |
| 1007.6932 | 4026.7439 | 4026.7313 | 96 | WQDMKNPGSMNTDPLFGYNSNDTNTDVGYPGGLFDK | 140 | 175 |
| 1103.1533 | 3306.4381 | 3306.4412 | 42 | NPGSMNTDPLFGYNSNDTNTDVGYPGGLFDK | 145 | 175 |
| 1108.4856 | 3322.435 | 3322.4361 | 65 | NPGSMNTDPLFGYNSNDTNTDVGYPGGLFDK | 145 | 175 |
| 1108.4857 | 3322.4353 | 3322.4361 | 49 | NPGSMNTDPLFGYNSNDTNTDVGYPGGLFDK | 145 | 175 |
| 831.6178 | 3322.4421 | 3322.4361 | 33 | NPGSMNTDPLFGYNSNDTNTDVGYPGGLFDK | 145 | 175 |
| 831.6181 | 3322.4433 | 3322.4361 | 19 | NPGSMNTDPLFGYNSNDTNTDVGYPGGLFDK | 145 | 175 |
| 481.7682 | 961.5218 | 961.5233 | 51 | LGYAKDPAK | 176 | 184 |

| Lhca3 | | | | | | |
| --- | --- | --- | --- | --- | --- | --- |
| Observed | Mr(expt) | Mr(calc) | Score | Peptide sequence | Amino acid position  start | Amino acid position  end |
| 627.3228 | 1252.6311 | 1252.6299 | 75 | AEEAEAAPAPVAK | 26 | 38 |
| 627.3229 | 1252.6313 | 1252.6299 | 65 | AEEAEAAPAPVAK | 26 | 38 |
| 546.9653 | 1637.8742 | 1637.8736 | 44 | AEEAEAAPAPVAKKTR | 26 | 41 |
| 448.9062 | 1343.6969 | 1343.6986 | 29 | WLAYSELIHGR | 91 | 101 |
| 448.9063 | 1343.697 | 1343.6986 | 28 | WLAYSELIHGR | 91 | 101 |
| 672.8558 | 1343.6971 | 1343.6986 | 19 | WLAYSELIHGR | 91 | 101 |
| 448.9066 | 1343.6981 | 1343.6986 | 21 | WLAYSELIHGR | 91 | 101 |
| 672.8568 | 1343.6991 | 1343.6986 | 20 | WLAYSELIHGR | 91 | 101 |
| 672.8572 | 1343.6999 | 1343.6986 | 57 | WLAYSELIHGR | 91 | 101 |
| 823.1763 | 3288.6762 | 3288.6749 | 31 | WAMLGVAGMVAPEVLGGMGIIPQETGLVWFK | 102 | 132 |
| 1097.2332 | 3288.6776 | 3288.6749 | 19 | WAMLGVAGMVAPEVLGGMGIIPQETGLVWFK | 102 | 132 |
| 1102.5647 | 3304.6723 | 3304.6699 | 63 | WAMLGVAGMVAPEVLGGMGIIPQETGLVWFK | 102 | 132 |
| 763.3468 | 1524.679 | 1524.6779 | 56 | RAQDYWNPGSMGK | 166 | 178 |
| 677.2981 | 1352.5816 | 1352.5819 | 56 | AQDYWNPGSMGK | 167 | 178 |
| 685.2926 | 1368.5706 | 1368.5768 | 70 | AQDYWNPGSMGK | 167 | 178 |
| 685.2941 | 1368.5736 | 1368.5768 | 22 | AQDYWNPGSMGK | 167 | 178 |
| 685.2957 | 1368.5769 | 1368.5768 | 70 | AQDYWNPGSMGK | 167 | 178 |
| 454.2296 | 906.4446 | 906.4447 | 38 | QDFAGLEK | 179 | 186 |
| 454.2296 | 906.4447 | 906.4447 | 54 | QDFAGLEK | 179 | 186 |
| 454.2302 | 906.4459 | 906.4447 | 42 | QDFAGLEK | 179 | 186 |
| 996.9531 | 1991.8917 | 1991.891 | 46 | MLGGSGDPAYPGGFFNFMK | 187 | 205 |
| 1004.9498 | 2007.885 | 2007.8859 | 46 | MLGGSGDPAYPGGFFNFMK | 187 | 205 |
| 1004.9504 | 2007.8863 | 2007.8859 | 50 | MLGGSGDPAYPGGFFNFMK | 187 | 205 |
| 675.6349 | 2023.8828 | 2023.8808 | 36 | MLGGSGDPAYPGGFFNFMK | 187 | 205 |
| 1012.9487 | 2023.8829 | 2023.8808 | 74 | MLGGSGDPAYPGGFFNFMK | 187 | 205 |
| 1012.9488 | 2023.8831 | 2023.8808 | 43 | MLGGSGDPAYPGGFFNFMK | 187 | 205 |
| 1012.9489 | 2023.8833 | 2023.8808 | 20 | MLGGSGDPAYPGGFFNFMK | 187 | 205 |
| 370.1752 | 1107.5038 | 1107.5052 | 32 | QGEKDMAAMK | 206 | 215 |
| 798.9957 | 2393.9652 | 2393.9605 | 64 | LAMMACFGCGAQACMTGEGPVK | 224 | 245 |
| 979.1756 | 2934.505 | 2934.5029 | 58 | NLVDHVIDPFGHNLLVNFSQIGGVSPF | 246 | 272 |
| 979.1785 | 2934.5136 | 2934.5029 | 42 | NLVDHVIDPFGHNLLVNFSQIGGVSPF | 246 | 272 |

| Lhca4 | | | | | | |
| --- | --- | --- | --- | --- | --- | --- |
| Observed | Mr(expt) | Mr(calc) | Score | Peptide sequence | Amino acid position  start | Amino acid position  end |
| 691.9899 | 2072.9478 | 2072.9448 | 23 | ASTQPMWFPGMDAPQHLK | 30 | 47 |
| 846.4163 | 1690.818 | 1690.8203 | 49 | GELPGDYGFDPLNLGK | 48 | 63 |
| 846.4163 | 1690.8181 | 1690.8203 | 46 | GELPGDYGFDPLNLGK | 48 | 63 |
| 846.4164 | 1690.8183 | 1690.8203 | 56 | GELPGDYGFDPLNLGK | 48 | 63 |
| 846.4165 | 1690.8184 | 1690.8203 | 43 | GELPGDYGFDPLNLGK | 48 | 63 |
| 846.4168 | 1690.8189 | 1690.8203 | 53 | GELPGDYGFDPLNLGK | 48 | 63 |
| 564.614 | 1690.82 | 1690.8203 | 50 | GELPGDYGFDPLNLGK | 48 | 63 |
| 846.4173 | 1690.82 | 1690.8203 | 52 | GELPGDYGFDPLNLGK | 48 | 63 |
| 846.4175 | 1690.8204 | 1690.8203 | 45 | GELPGDYGFDPLNLGK | 48 | 63 |
| 846.4177 | 1690.8208 | 1690.8203 | 69 | GELPGDYGFDPLNLGK | 48 | 63 |
| 846.4177 | 1690.8209 | 1690.8203 | 45 | GELPGDYGFDPLNLGK | 48 | 63 |
| 846.4177 | 1690.8209 | 1690.8203 | 69 | GELPGDYGFDPLNLGK | 48 | 63 |
| 846.4178 | 1690.821 | 1690.8203 | 64 | GELPGDYGFDPLNLGK | 48 | 63 |
| 846.4178 | 1690.8211 | 1690.8203 | 60 | GELPGDYGFDPLNLGK | 48 | 63 |
| 846.4179 | 1690.8213 | 1690.8203 | 51 | GELPGDYGFDPLNLGK | 48 | 63 |
| 846.418 | 1690.8214 | 1690.8203 | 70 | GELPGDYGFDPLNLGK | 48 | 63 |
| 846.418 | 1690.8215 | 1690.8203 | 36 | GELPGDYGFDPLNLGK | 48 | 63 |
| 846.4182 | 1690.8219 | 1690.8203 | 44 | GELPGDYGFDPLNLGK | 48 | 63 |
| 846.4182 | 1690.8219 | 1690.8203 | 64 | GELPGDYGFDPLNLGK | 48 | 63 |
| 846.4183 | 1690.8221 | 1690.8203 | 45 | GELPGDYGFDPLNLGK | 48 | 63 |
| 846.4186 | 1690.8227 | 1690.8203 | 51 | GELPGDYGFDPLNLGK | 48 | 63 |
| 846.4191 | 1690.8236 | 1690.8203 | 61 | GELPGDYGFDPLNLGK | 48 | 63 |
| 846.4195 | 1690.8244 | 1690.8203 | 51 | GELPGDYGFDPLNLGK | 48 | 63 |
| 682.6766 | 2045.0079 | 2045.0106 | 22 | GELPGDYGFDPLNLGKEPK | 48 | 66 |
| 1023.5131 | 2045.0116 | 2045.0106 | 57 | GELPGDYGFDPLNLGKEPK | 48 | 66 |
| 581.9527 | 1742.8363 | 1742.8376 | 22 | DLEWYVQAELQHGR | 67 | 80 |
| 872.4265 | 1742.8385 | 1742.8376 | 69 | DLEWYVQAELQHGR | 67 | 80 |
| 581.9536 | 1742.839 | 1742.8376 | 27 | DLEWYVQAELQHGR | 67 | 80 |
| 849.9634 | 1697.9122 | 1697.9174 | 64 | WAMLGVAGAAAPEILTK | 81 | 97 |
| 849.9646 | 1697.9146 | 1697.9174 | 56 | WAMLGVAGAAAPEILTK | 81 | 97 |
| 849.9654 | 1697.9163 | 1697.9174 | 47 | WAMLGVAGAAAPEILTK | 81 | 97 |
| 849.9659 | 1697.9173 | 1697.9174 | 31 | WAMLGVAGAAAPEILTK | 81 | 97 |
| 849.966 | 1697.9174 | 1697.9174 | 58 | WAMLGVAGAAAPEILTK | 81 | 97 |
| 849.9661 | 1697.9176 | 1697.9174 | 66 | WAMLGVAGAAAPEILTK | 81 | 97 |
| 849.9661 | 1697.9176 | 1697.9174 | 74 | WAMLGVAGAAAPEILTK | 81 | 97 |
| 566.9799 | 1697.9179 | 1697.9174 | 58 | WAMLGVAGAAAPEILTK | 81 | 97 |
| 849.9663 | 1697.9181 | 1697.9174 | 55 | WAMLGVAGAAAPEILTK | 81 | 97 |
| 849.9666 | 1697.9185 | 1697.9174 | 73 | WAMLGVAGAAAPEILTK | 81 | 97 |
| 849.9666 | 1697.9187 | 1697.9174 | 72 | WAMLGVAGAAAPEILTK | 81 | 97 |
| 849.9667 | 1697.9188 | 1697.9174 | 77 | WAMLGVAGAAAPEILTK | 81 | 97 |
| 849.9669 | 1697.9192 | 1697.9174 | 46 | WAMLGVAGAAAPEILTK | 81 | 97 |
| 849.9675 | 1697.9205 | 1697.9174 | 52 | WAMLGVAGAAAPEILTK | 81 | 97 |
| 849.9675 | 1697.9205 | 1697.9174 | 88 | WAMLGVAGAAAPEILTK | 81 | 97 |
| 857.9631 | 1713.9117 | 1713.9124 | 74 | WAMLGVAGAAAPEILTK | 81 | 97 |
| 857.9633 | 1713.912 | 1713.9124 | 85 | WAMLGVAGAAAPEILTK | 81 | 97 |
| 857.9633 | 1713.9121 | 1713.9124 | 106 | WAMLGVAGAAAPEILTK | 81 | 97 |
| 857.9635 | 1713.9124 | 1713.9124 | 32 | WAMLGVAGAAAPEILTK | 81 | 97 |
| 857.9639 | 1713.9132 | 1713.9124 | 84 | WAMLGVAGAAAPEILTK | 81 | 97 |
| 857.9639 | 1713.9132 | 1713.9124 | 46 | WAMLGVAGAAAPEILTK | 81 | 97 |
| 857.9639 | 1713.9133 | 1713.9124 | 104 | WAMLGVAGAAAPEILTK | 81 | 97 |
| 857.9639 | 1713.9133 | 1713.9124 | 79 | WAMLGVAGAAAPEILTK | 81 | 97 |
| 572.3118 | 1713.9135 | 1713.9124 | 54 | WAMLGVAGAAAPEILTK | 81 | 97 |
| 857.9642 | 1713.9139 | 1713.9124 | 75 | WAMLGVAGAAAPEILTK | 81 | 97 |
| 857.9645 | 1713.9144 | 1713.9124 | 45 | WAMLGVAGAAAPEILTK | 81 | 97 |
| 857.9653 | 1713.9161 | 1713.9124 | 48 | WAMLGVAGAAAPEILTK | 81 | 97 |
| 857.9653 | 1713.9161 | 1713.9124 | 44 | WAMLGVAGAAAPEILTK | 81 | 97 |
| 773.8778 | 1545.7411 | 1545.7423 | 61 | KPGSVNEDPAFSGNK | 143 | 157 |
| 516.2546 | 1545.7421 | 1545.7423 | 30 | KPGSVNEDPAFSGNK | 143 | 157 |
| 516.2549 | 1545.743 | 1545.7423 | 26 | KPGSVNEDPAFSGNK | 143 | 157 |
| 773.8788 | 1545.743 | 1545.7423 | 75 | KPGSVNEDPAFSGNK | 143 | 157 |
| 773.879 | 1545.7434 | 1545.7423 | 77 | KPGSVNEDPAFSGNK | 143 | 157 |
| 887.9569 | 3547.7985 | 3547.7987 | 35 | KPGSVNEDPAFSGNKLPSGIVGYPGGIFDPLGYAK | 143 | 177 |
| 1183.6083 | 3547.803 | 3547.7987 | 87 | KPGSVNEDPAFSGNKLPSGIVGYPGGIFDPLGYAK | 143 | 177 |
| 674.3628 | 2020.0665 | 2020.067 | 51 | LPSGIVGYPGGIFDPLGYAK | 158 | 177 |
| 1011.041 | 2020.0675 | 2020.067 | 62 | LPSGIVGYPGGIFDPLGYAK | 158 | 177 |
| 1011.0412 | 2020.0678 | 2020.067 | 61 | LPSGIVGYPGGIFDPLGYAK | 158 | 177 |
| 1011.0413 | 2020.068 | 2020.067 | 31 | LPSGIVGYPGGIFDPLGYAK | 158 | 177 |
| 674.3633 | 2020.068 | 2020.067 | 36 | LPSGIVGYPGGIFDPLGYAK | 158 | 177 |
| 1011.0414 | 2020.0682 | 2020.067 | 37 | LPSGIVGYPGGIFDPLGYAK | 158 | 177 |
| 1011.0414 | 2020.0683 | 2020.067 | 63 | LPSGIVGYPGGIFDPLGYAK | 158 | 177 |
| 1011.0415 | 2020.0684 | 2020.067 | 60 | LPSGIVGYPGGIFDPLGYAK | 158 | 177 |
| 1011.0415 | 2020.0684 | 2020.067 | 30 | LPSGIVGYPGGIFDPLGYAK | 158 | 177 |
| 1011.0415 | 2020.0684 | 2020.067 | 38 | LPSGIVGYPGGIFDPLGYAK | 158 | 177 |
| 1011.0416 | 2020.0686 | 2020.067 | 36 | LPSGIVGYPGGIFDPLGYAK | 158 | 177 |
| 1011.0416 | 2020.0686 | 2020.067 | 49 | LPSGIVGYPGGIFDPLGYAK | 158 | 177 |
| 1011.0416 | 2020.0687 | 2020.067 | 44 | LPSGIVGYPGGIFDPLGYAK | 158 | 177 |
| 674.3635 | 2020.0688 | 2020.067 | 73 | LPSGIVGYPGGIFDPLGYAK | 158 | 177 |
| 674.3635 | 2020.0688 | 2020.067 | 15 | LPSGIVGYPGGIFDPLGYAK | 158 | 177 |
| 1011.0418 | 2020.0691 | 2020.067 | 67 | LPSGIVGYPGGIFDPLGYAK | 158 | 177 |
| 674.3637 | 2020.0693 | 2020.067 | 28 | LPSGIVGYPGGIFDPLGYAK | 158 | 177 |
| 1011.0419 | 2020.0693 | 2020.067 | 51 | LPSGIVGYPGGIFDPLGYAK | 158 | 177 |
| 674.3638 | 2020.0697 | 2020.067 | 30 | LPSGIVGYPGGIFDPLGYAK | 158 | 177 |
| 1011.0421 | 2020.0697 | 2020.067 | 39 | LPSGIVGYPGGIFDPLGYAK | 158 | 177 |
| 1011.0428 | 2020.0711 | 2020.067 | 38 | LPSGIVGYPGGIFDPLGYAK | 158 | 177 |
| 674.3644 | 2020.0713 | 2020.067 | 18 | LPSGIVGYPGGIFDPLGYAK | 158 | 177 |

| Lhca5 | | | | | | |
| --- | --- | --- | --- | --- | --- | --- |
| Observed | Mr(expt) | Mr(calc) | Score | Peptide sequence | Amino acid position  start | Amino acid position  end |
| 383.8758 | 1148.6055 | 1148.6052 | 21 | KMWLPAPYK | 32 | 40 |
| 575.3108 | 1148.607 | 1148.6052 | 24 | KMWLPAPYK | 32 | 40 |
| 503.265 | 1004.5153 | 1004.5153 | 27 | MWLPAPYK | 33 | 40 |
| 511.2614 | 1020.5083 | 1020.5103 | 20 | MWLPAPYK | 33 | 40 |
| 511.262 | 1020.5094 | 1020.5103 | 27 | MWLPAPYK | 33 | 40 |
| 511.2624 | 1020.5103 | 1020.5103 | 22 | MWLPAPYK | 33 | 40 |
| 657.3165 | 2625.237 | 2625.246 | 34 | APAHLDGSIAGDYGFDPLGLGTNPDR | 41 | 66 |
| 876.0872 | 2625.2398 | 2625.246 | 30 | APAHLDGSIAGDYGFDPLGLGTNPDR | 41 | 66 |
| 876.088 | 2625.242 | 2625.246 | 24 | APAHLDGSIAGDYGFDPLGLGTNPDR | 41 | 66 |
| 876.088 | 2625.2422 | 2625.246 | 55 | APAHLDGSIAGDYGFDPLGLGTNPDR | 41 | 66 |
| 876.0885 | 2625.2437 | 2625.246 | 26 | APAHLDGSIAGDYGFDPLGLGTNPDR | 41 | 66 |
| 876.0886 | 2625.244 | 2625.246 | 45 | APAHLDGSIAGDYGFDPLGLGTNPDR | 41 | 66 |
| 876.0886 | 2625.244 | 2625.246 | 36 | APAHLDGSIAGDYGFDPLGLGTNPDR | 41 | 66 |
| 876.0888 | 2625.2444 | 2625.246 | 40 | APAHLDGSIAGDYGFDPLGLGTNPDR | 41 | 66 |
| 876.0888 | 2625.2446 | 2625.246 | 47 | APAHLDGSIAGDYGFDPLGLGTNPDR | 41 | 66 |
| 876.0889 | 2625.245 | 2625.246 | 52 | APAHLDGSIAGDYGFDPLGLGTNPDR | 41 | 66 |
| 876.0889 | 2625.245 | 2625.246 | 43 | APAHLDGSIAGDYGFDPLGLGTNPDR | 41 | 66 |
| 876.0892 | 2625.2459 | 2625.246 | 36 | APAHLDGSIAGDYGFDPLGLGTNPDR | 41 | 66 |
| 876.0895 | 2625.2468 | 2625.246 | 34 | APAHLDGSIAGDYGFDPLGLGTNPDR | 41 | 66 |
| 876.0896 | 2625.247 | 2625.246 | 61 | APAHLDGSIAGDYGFDPLGLGTNPDR | 41 | 66 |
| 876.0899 | 2625.2479 | 2625.246 | 40 | APAHLDGSIAGDYGFDPLGLGTNPDR | 41 | 66 |
| 876.09 | 2625.2483 | 2625.246 | 43 | APAHLDGSIAGDYGFDPLGLGTNPDR | 41 | 66 |
| 876.0901 | 2625.2484 | 2625.246 | 44 | APAHLDGSIAGDYGFDPLGLGTNPDR | 41 | 66 |
| 876.0901 | 2625.2484 | 2625.246 | 49 | APAHLDGSIAGDYGFDPLGLGTNPDR | 41 | 66 |
| 876.0902 | 2625.2488 | 2625.246 | 34 | APAHLDGSIAGDYGFDPLGLGTNPDR | 41 | 66 |
| 876.0904 | 2625.2495 | 2625.246 | 35 | APAHLDGSIAGDYGFDPLGLGTNPDR | 41 | 66 |
| 876.0906 | 2625.2501 | 2625.246 | 30 | APAHLDGSIAGDYGFDPLGLGTNPDR | 41 | 66 |
| 876.0907 | 2625.2503 | 2625.246 | 33 | APAHLDGSIAGDYGFDPLGLGTNPDR | 41 | 66 |
| 876.0908 | 2625.2505 | 2625.246 | 44 | APAHLDGSIAGDYGFDPLGLGTNPDR | 41 | 66 |
| 1313.6329 | 2625.2513 | 2625.246 | 71 | APAHLDGSIAGDYGFDPLGLGTNPDR | 41 | 66 |
| 876.0924 | 2625.2554 | 2625.246 | 45 | APAHLDGSIAGDYGFDPLGLGTNPDR | 41 | 66 |
| 717.6142 | 2866.4277 | 2866.425 | 27 | APAHLDGSIAGDYGFDPLGLGTNPDRLK | 41 | 68 |
| 717.6153 | 2866.4321 | 2866.425 | 24 | APAHLDGSIAGDYGFDPLGLGTNPDRLK | 41 | 68 |
| 822.9066 | 1643.7986 | 1643.7977 | 34 | LKYYQEAELMNAR | 67 | 79 |
| 694.32 | 1386.6255 | 1386.6237 | 84 | YYQEAELMNAR | 69 | 79 |
| 702.3152 | 1402.6158 | 1402.6187 | 42 | YYQEAELMNAR | 69 | 79 |
| 702.3155 | 1402.6164 | 1402.6187 | 51 | YYQEAELMNAR | 69 | 79 |
| 702.3168 | 1402.6191 | 1402.6187 | 58 | YYQEAELMNAR | 69 | 79 |
| 702.317 | 1402.6195 | 1402.6187 | 41 | YYQEAELMNAR | 69 | 79 |
| 702.3172 | 1402.6198 | 1402.6187 | 57 | YYQEAELMNAR | 69 | 79 |
| 468.5476 | 1402.6209 | 1402.6187 | 42 | YYQEAELMNAR | 69 | 79 |
| 702.3178 | 1402.6211 | 1402.6187 | 61 | YYQEAELMNAR | 69 | 79 |
| 702.3184 | 1402.6222 | 1402.6187 | 47 | YYQEAELMNAR | 69 | 79 |
| 686.6872 | 2057.0397 | 2057.0438 | 38 | WAMMAVAGIVGTEVAGIEPR | 80 | 99 |
| 686.6879 | 2057.042 | 2057.0438 | 67 | WAMMAVAGIVGTEVAGIEPR | 80 | 99 |
| 1029.5288 | 2057.0431 | 2057.0438 | 17 | WAMMAVAGIVGTEVAGIEPR | 80 | 99 |
| 686.6885 | 2057.0436 | 2057.0438 | 42 | WAMMAVAGIVGTEVAGIEPR | 80 | 99 |
| 686.6888 | 2057.0445 | 2057.0438 | 36 | WAMMAVAGIVGTEVAGIEPR | 80 | 99 |
| 686.6889 | 2057.0449 | 2057.0438 | 25 | WAMMAVAGIVGTEVAGIEPR | 80 | 99 |
| 686.6892 | 2057.0456 | 2057.0438 | 31 | WAMMAVAGIVGTEVAGIEPR | 80 | 99 |
| 686.6892 | 2057.0458 | 2057.0438 | 43 | WAMMAVAGIVGTEVAGIEPR | 80 | 99 |
| 686.6895 | 2057.0467 | 2057.0438 | 59 | WAMMAVAGIVGTEVAGIEPR | 80 | 99 |
| 686.6898 | 2057.0476 | 2057.0438 | 25 | WAMMAVAGIVGTEVAGIEPR | 80 | 99 |
| 686.6899 | 2057.0478 | 2057.0438 | 54 | WAMMAVAGIVGTEVAGIEPR | 80 | 99 |
| 1029.5331 | 2057.0516 | 2057.0438 | 16 | WAMMAVAGIVGTEVAGIEPR | 80 | 99 |
| 692.0176 | 2073.0309 | 2073.0387 | 40 | WAMMAVAGIVGTEVAGIEPR | 80 | 99 |
| 692.0187 | 2073.0344 | 2073.0387 | 51 | WAMMAVAGIVGTEVAGIEPR | 80 | 99 |
| 692.019 | 2073.0353 | 2073.0387 | 44 | WAMMAVAGIVGTEVAGIEPR | 80 | 99 |
| 692.0195 | 2073.0366 | 2073.0387 | 55 | WAMMAVAGIVGTEVAGIEPR | 80 | 99 |
| 692.0196 | 2073.0369 | 2073.0387 | 44 | WAMMAVAGIVGTEVAGIEPR | 80 | 99 |
| 692.0197 | 2073.0373 | 2073.0387 | 44 | WAMMAVAGIVGTEVAGIEPR | 80 | 99 |
| 1037.5261 | 2073.0377 | 2073.0387 | 47 | WAMMAVAGIVGTEVAGIEPR | 80 | 99 |
| 692.0199 | 2073.0379 | 2073.0387 | 24 | WAMMAVAGIVGTEVAGIEPR | 80 | 99 |
| 692.0199 | 2073.0379 | 2073.0387 | 46 | WAMMAVAGIVGTEVAGIEPR | 80 | 99 |
| 1037.5262 | 2073.0379 | 2073.0387 | 73 | WAMMAVAGIVGTEVAGIEPR | 80 | 99 |
| 692.02 | 2073.0381 | 2073.0387 | 76 | WAMMAVAGIVGTEVAGIEPR | 80 | 99 |
| 692.02 | 2073.0381 | 2073.0387 | 50 | WAMMAVAGIVGTEVAGIEPR | 80 | 99 |
| 692.0201 | 2073.0386 | 2073.0387 | 36 | WAMMAVAGIVGTEVAGIEPR | 80 | 99 |
| 1037.5266 | 2073.0387 | 2073.0387 | 67 | WAMMAVAGIVGTEVAGIEPR | 80 | 99 |
| 692.0202 | 2073.0388 | 2073.0387 | 56 | WAMMAVAGIVGTEVAGIEPR | 80 | 99 |
| 692.0202 | 2073.0388 | 2073.0387 | 64 | WAMMAVAGIVGTEVAGIEPR | 80 | 99 |
| 692.0203 | 2073.039 | 2073.0387 | 47 | WAMMAVAGIVGTEVAGIEPR | 80 | 99 |
| 692.0203 | 2073.0391 | 2073.0387 | 47 | WAMMAVAGIVGTEVAGIEPR | 80 | 99 |
| 1037.5275 | 2073.0404 | 2073.0387 | 39 | WAMMAVAGIVGTEVAGIEPR | 80 | 99 |
| 692.0209 | 2073.0408 | 2073.0387 | 53 | WAMMAVAGIVGTEVAGIEPR | 80 | 99 |
| 692.021 | 2073.0412 | 2073.0387 | 54 | WAMMAVAGIVGTEVAGIEPR | 80 | 99 |
| 1037.5289 | 2073.0433 | 2073.0387 | 76 | WAMMAVAGIVGTEVAGIEPR | 80 | 99 |
| 692.0218 | 2073.0437 | 2073.0387 | 50 | WAMMAVAGIVGTEVAGIEPR | 80 | 99 |
| 1037.5299 | 2073.0453 | 2073.0387 | 29 | WAMMAVAGIVGTEVAGIEPR | 80 | 99 |
| 1045.5216 | 2089.0287 | 2089.0336 | 81 | WAMMAVAGIVGTEVAGIEPR | 80 | 99 |
| 697.3502 | 2089.0288 | 2089.0336 | 67 | WAMMAVAGIVGTEVAGIEPR | 80 | 99 |
| 697.3503 | 2089.0292 | 2089.0336 | 69 | WAMMAVAGIVGTEVAGIEPR | 80 | 99 |
| 697.3505 | 2089.0297 | 2089.0336 | 56 | WAMMAVAGIVGTEVAGIEPR | 80 | 99 |
| 697.3507 | 2089.0303 | 2089.0336 | 45 | WAMMAVAGIVGTEVAGIEPR | 80 | 99 |
| 1045.5225 | 2089.0304 | 2089.0336 | 32 | WAMMAVAGIVGTEVAGIEPR | 80 | 99 |
| 697.3509 | 2089.0308 | 2089.0336 | 50 | WAMMAVAGIVGTEVAGIEPR | 80 | 99 |
| 697.3511 | 2089.0316 | 2089.0336 | 65 | WAMMAVAGIVGTEVAGIEPR | 80 | 99 |
| 697.3513 | 2089.032 | 2089.0336 | 50 | WAMMAVAGIVGTEVAGIEPR | 80 | 99 |
| 697.3513 | 2089.0321 | 2089.0336 | 51 | WAMMAVAGIVGTEVAGIEPR | 80 | 99 |
| 1045.5234 | 2089.0323 | 2089.0336 | 85 | WAMMAVAGIVGTEVAGIEPR | 80 | 99 |
| 697.3514 | 2089.0325 | 2089.0336 | 68 | WAMMAVAGIVGTEVAGIEPR | 80 | 99 |
| 697.3516 | 2089.0329 | 2089.0336 | 45 | WAMMAVAGIVGTEVAGIEPR | 80 | 99 |
| 1045.5238 | 2089.033 | 2089.0336 | 40 | WAMMAVAGIVGTEVAGIEPR | 80 | 99 |
| 697.3517 | 2089.0332 | 2089.0336 | 44 | WAMMAVAGIVGTEVAGIEPR | 80 | 99 |
| 1045.5239 | 2089.0333 | 2089.0336 | 38 | WAMMAVAGIVGTEVAGIEPR | 80 | 99 |
| 697.3518 | 2089.0334 | 2089.0336 | 51 | WAMMAVAGIVGTEVAGIEPR | 80 | 99 |
| 697.3519 | 2089.034 | 2089.0336 | 54 | WAMMAVAGIVGTEVAGIEPR | 80 | 99 |
| 697.3522 | 2089.0347 | 2089.0336 | 53 | WAMMAVAGIVGTEVAGIEPR | 80 | 99 |
| 697.3522 | 2089.0349 | 2089.0336 | 46 | WAMMAVAGIVGTEVAGIEPR | 80 | 99 |
| 697.3522 | 2089.0349 | 2089.0336 | 44 | WAMMAVAGIVGTEVAGIEPR | 80 | 99 |
| 697.3524 | 2089.0353 | 2089.0336 | 58 | WAMMAVAGIVGTEVAGIEPR | 80 | 99 |
| 697.3524 | 2089.0354 | 2089.0336 | 49 | WAMMAVAGIVGTEVAGIEPR | 80 | 99 |
| 697.3524 | 2089.0354 | 2089.0336 | 38 | WAMMAVAGIVGTEVAGIEPR | 80 | 99 |
| 697.3524 | 2089.0354 | 2089.0336 | 39 | WAMMAVAGIVGTEVAGIEPR | 80 | 99 |
| 697.3525 | 2089.0356 | 2089.0336 | 39 | WAMMAVAGIVGTEVAGIEPR | 80 | 99 |
| 697.3533 | 2089.0382 | 2089.0336 | 38 | WAMMAVAGIVGTEVAGIEPR | 80 | 99 |
| 697.3539 | 2089.04 | 2089.0336 | 44 | WAMMAVAGIVGTEVAGIEPR | 80 | 99 |
| 1143.8898 | 3428.6475 | 3428.6427 | 62 | WWEAGTEDYGFPPAALLAIQFPVMGYLENK | 100 | 129 |
| 1195.919 | 3584.735 | 3584.7438 | 108 | WWEAGTEDYGFPPAALLAIQFPVMGYLENKR | 100 | 130 |
| 897.1928 | 3584.7419 | 3584.7438 | 51 | WWEAGTEDYGFPPAALLAIQFPVMGYLENKR | 100 | 130 |
| 1195.9243 | 3584.7511 | 3584.7438 | 110 | WWEAGTEDYGFPPAALLAIQFPVMGYLENKR | 100 | 130 |
| 974.1075 | 2919.3008 | 2919.299 | 29 | RIQGWMATGSSGVNETFPFDPMGMGSK | 130 | 156 |
| 979.4384 | 2935.2934 | 2935.2939 | 25 | RIQGWMATGSSGVNETFPFDPMGMGSK | 130 | 156 |
| 979.4397 | 2935.2973 | 2935.2939 | 70 | RIQGWMATGSSGVNETFPFDPMGMGSK | 130 | 156 |
| 916.7413 | 2747.202 | 2747.203 | 32 | IQGWMATGSSGVNETFPFDPMGMGSK | 131 | 156 |
| 916.7413 | 2747.2022 | 2747.203 | 26 | IQGWMATGSSGVNETFPFDPMGMGSK | 131 | 156 |
| 922.0737 | 2763.1992 | 2763.1979 | 58 | IQGWMATGSSGVNETFPFDPMGMGSK | 131 | 156 |
| 922.0743 | 2763.2012 | 2763.1979 | 53 | IQGWMATGSSGVNETFPFDPMGMGSK | 131 | 156 |
| 927.4052 | 2779.1936 | 2779.1928 | 37 | IQGWMATGSSGVNETFPFDPMGMGSK | 131 | 156 |
| 927.4057 | 2779.1953 | 2779.1928 | 54 | IQGWMATGSSGVNETFPFDPMGMGSK | 131 | 156 |
| 927.407 | 2779.1991 | 2779.1928 | 34 | IQGWMATGSSGVNETFPFDPMGMGSK | 131 | 156 |
| 868.1652 | 2601.4738 | 2601.4716 | 44 | AAMIAFVGIVVQAIVYREGPVAALK | 170 | 194 |
| 392.7316 | 783.4487 | 783.449 | 23 | EGPVAALK | 187 | 194 |
| 392.7323 | 783.45 | 783.449 | 34 | EGPVAALK | 187 | 194 |
| 392.7324 | 783.4503 | 783.449 | 37 | EGPVAALK | 187 | 194 |
| 848.3996 | 2542.177 | 2542.1767 | 32 | DHISNPFGCNMATNIMNIPVNLA | 195 | 217 |
| 1272.0991 | 2542.1837 | 2542.1767 | 41 | DHISNPFGCNMATNIMNIPVNLA | 195 | 217 |
| 853.7294 | 2558.1663 | 2558.1716 | 39 | DHISNPFGCNMATNIMNIPVNLA | 195 | 217 |
| 853.7308 | 2558.1705 | 2558.1716 | 46 | DHISNPFGCNMATNIMNIPVNLA | 195 | 217 |
| 1280.0933 | 2558.172 | 2558.1716 | 48 | DHISNPFGCNMATNIMNIPVNLA | 195 | 217 |
| 1280.0956 | 2558.1766 | 2558.1716 | 55 | DHISNPFGCNMATNIMNIPVNLA | 195 | 217 |
| 859.0636 | 2574.169 | 2574.1665 | 30 | DHISNPFGCNMATNIMNIPVNLA | 195 | 217 |
| 1288.0938 | 2574.1729 | 2574.1665 | 51 | DHISNPFGCNMATNIMNIPVNLA | 195 | 217 |

| Lhca6 | | | | | | |
| --- | --- | --- | --- | --- | --- | --- |
| Observed | Mr(expt) | Mr(calc) | Score | Peptide sequence | Amino acid position  start | Amino acid position  end |
| 740.0443 | 2217.1111 | 2217.1099 | 66 | LGANVETLPYLQEAELMNGR | 12 | 31 |
| 1109.563 | 2217.1114 | 2217.1099 | 70 | LGANVETLPYLQEAELMNGR | 12 | 31 |
| 745.3743 | 2233.101 | 2233.1048 | 50 | LGANVETLPYLQEAELMNGR | 12 | 31 |
| 745.3745 | 2233.1017 | 2233.1048 | 69 | LGANVETLPYLQEAELMNGR | 12 | 31 |
| 745.3766 | 2233.1079 | 2233.1048 | 44 | LGANVETLPYLQEAELMNGR | 12 | 31 |
| 1117.5625 | 2233.1104 | 2233.1048 | 49 | LGANVETLPYLQEAELMNGR | 12 | 31 |
| 1117.5652 | 2233.1158 | 2233.1048 | 70 | LGANVETLPYLQEAELMNGR | 12 | 31 |
| 968.0046 | 1933.9947 | 1933.9972 | 65 | WAMAATAGILFTDATGLPK | 32 | 50 |
| 645.6727 | 1933.9964 | 1933.9972 | 22 | WAMAATAGILFTDATGLPK | 32 | 50 |
| 968.0055 | 1933.9964 | 1933.9972 | 68 | WAMAATAGILFTDATGLPK | 32 | 50 |
| 645.6728 | 1933.9967 | 1933.9972 | 35 | WAMAATAGILFTDATGLPK | 32 | 50 |
| 968.0059 | 1933.9973 | 1933.9972 | 83 | WAMAATAGILFTDATGLPK | 32 | 50 |
| 645.6731 | 1933.9975 | 1933.9972 | 74 | WAMAATAGILFTDATGLPK | 32 | 50 |
| 645.6733 | 1933.9982 | 1933.9972 | 71 | WAMAATAGILFTDATGLPK | 32 | 50 |
| 645.6734 | 1933.9984 | 1933.9972 | 22 | WAMAATAGILFTDATGLPK | 32 | 50 |
| 968.0068 | 1933.9991 | 1933.9972 | 51 | WAMAATAGILFTDATGLPK | 32 | 50 |
| 968.0074 | 1934.0002 | 1933.9972 | 52 | WAMAATAGILFTDATGLPK | 32 | 50 |
| 968.0076 | 1934.0006 | 1933.9972 | 56 | WAMAATAGILFTDATGLPK | 32 | 50 |
| 968.0087 | 1934.0029 | 1933.9972 | 59 | WAMAATAGILFTDATGLPK | 32 | 50 |
| 975.9991 | 1949.9836 | 1949.9921 | 68 | WAMAATAGILFTDATGLPK | 32 | 50 |
| 976 | 1949.9854 | 1949.9921 | 62 | WAMAATAGILFTDATGLPK | 32 | 50 |
| 976.0002 | 1949.9859 | 1949.9921 | 46 | WAMAATAGILFTDATGLPK | 32 | 50 |
| 976.0011 | 1949.9876 | 1949.9921 | 40 | WAMAATAGILFTDATGLPK | 32 | 50 |
| 976.0017 | 1949.9889 | 1949.9921 | 59 | WAMAATAGILFTDATGLPK | 32 | 50 |
| 651.0048 | 1949.9925 | 1949.9921 | 18 | WAMAATAGILFTDATGLPK | 32 | 50 |
| 976.0036 | 1949.9926 | 1949.9921 | 49 | WAMAATAGILFTDATGLPK | 32 | 50 |
| 976.0036 | 1949.9926 | 1949.9921 | 15 | WAMAATAGILFTDATGLPK | 32 | 50 |
| 651.005 | 1949.9932 | 1949.9921 | 56 | WAMAATAGILFTDATGLPK | 32 | 50 |
| 976.0043 | 1949.994 | 1949.9921 | 98 | WAMAATAGILFTDATGLPK | 32 | 50 |
| 651.0053 | 1949.9941 | 1949.9921 | 66 | WAMAATAGILFTDATGLPK | 32 | 50 |
| 976.0046 | 1949.9946 | 1949.9921 | 62 | WAMAATAGILFTDATGLPK | 32 | 50 |
| 976.0058 | 1949.997 | 1949.9921 | 61 | WAMAATAGILFTDATGLPK | 32 | 50 |
| 1139.5374 | 2277.0601 | 2277.0624 | 38 | TGESGVLNMFPFDPAGLDAPDK | 88 | 109 |
| 765.3602 | 2293.0587 | 2293.0573 | 57 | TGESGVLNMFPFDPAGLDAPDK | 88 | 109 |
| 1147.5375 | 2293.0604 | 2293.0573 | 76 | TGESGVLNMFPFDPAGLDAPDK | 88 | 109 |
| 812.0619 | 2433.1638 | 2433.1635 | 53 | TGESGVLNMFPFDPAGLDAPDKR | 88 | 110 |
| 812.0623 | 2433.165 | 2433.1635 | 62 | TGESGVLNMFPFDPAGLDAPDKR | 88 | 110 |
| 1217.5906 | 2433.1666 | 2433.1635 | 70 | TGESGVLNMFPFDPAGLDAPDKR | 88 | 110 |
| 812.0629 | 2433.1668 | 2433.1635 | 48 | TGESGVLNMFPFDPAGLDAPDKR | 88 | 110 |
| 817.3919 | 2449.1539 | 2449.1584 | 47 | TGESGVLNMFPFDPAGLDAPDKR | 88 | 110 |
| 817.3921 | 2449.1544 | 2449.1584 | 67 | TGESGVLNMFPFDPAGLDAPDKR | 88 | 110 |
| 817.3923 | 2449.1552 | 2449.1584 | 55 | TGESGVLNMFPFDPAGLDAPDKR | 88 | 110 |
| 817.3925 | 2449.1556 | 2449.1584 | 73 | TGESGVLNMFPFDPAGLDAPDKR | 88 | 110 |
| 817.393 | 2449.1572 | 2449.1584 | 60 | TGESGVLNMFPFDPAGLDAPDKR | 88 | 110 |
| 817.3931 | 2449.1574 | 2449.1584 | 60 | TGESGVLNMFPFDPAGLDAPDKR | 88 | 110 |
| 817.3932 | 2449.1579 | 2449.1584 | 54 | TGESGVLNMFPFDPAGLDAPDKR | 88 | 110 |
| 817.3932 | 2449.1579 | 2449.1584 | 84 | TGESGVLNMFPFDPAGLDAPDKR | 88 | 110 |
| 817.3934 | 2449.1583 | 2449.1584 | 61 | TGESGVLNMFPFDPAGLDAPDKR | 88 | 110 |
| 817.3935 | 2449.1586 | 2449.1584 | 62 | TGESGVLNMFPFDPAGLDAPDKR | 88 | 110 |
| 817.3935 | 2449.1586 | 2449.1584 | 79 | TGESGVLNMFPFDPAGLDAPDKR | 88 | 110 |
| 817.3936 | 2449.159 | 2449.1584 | 58 | TGESGVLNMFPFDPAGLDAPDKR | 88 | 110 |
| 817.3936 | 2449.159 | 2449.1584 | 77 | TGESGVLNMFPFDPAGLDAPDKR | 88 | 110 |
| 817.3937 | 2449.1592 | 2449.1584 | 72 | TGESGVLNMFPFDPAGLDAPDKR | 88 | 110 |
| 817.3939 | 2449.1599 | 2449.1584 | 57 | TGESGVLNMFPFDPAGLDAPDKR | 88 | 110 |
| 817.3939 | 2449.1599 | 2449.1584 | 68 | TGESGVLNMFPFDPAGLDAPDKR | 88 | 110 |
| 1225.5873 | 2449.16 | 2449.1584 | 48 | TGESGVLNMFPFDPAGLDAPDKR | 88 | 110 |
| 817.3942 | 2449.1607 | 2449.1584 | 66 | TGESGVLNMFPFDPAGLDAPDKR | 88 | 110 |
| 817.3942 | 2449.1609 | 2449.1584 | 73 | TGESGVLNMFPFDPAGLDAPDKR | 88 | 110 |
| 817.3942 | 2449.1609 | 2449.1584 | 68 | TGESGVLNMFPFDPAGLDAPDKR | 88 | 110 |
| 817.3943 | 2449.161 | 2449.1584 | 64 | TGESGVLNMFPFDPAGLDAPDKR | 88 | 110 |
| 817.3944 | 2449.1614 | 2449.1584 | 58 | TGESGVLNMFPFDPAGLDAPDKR | 88 | 110 |
| 817.3948 | 2449.1625 | 2449.1584 | 79 | TGESGVLNMFPFDPAGLDAPDKR | 88 | 110 |
| 817.3949 | 2449.1629 | 2449.1584 | 15 | TGESGVLNMFPFDPAGLDAPDKR | 88 | 110 |
| 817.3949 | 2449.1629 | 2449.1584 | 78 | TGESGVLNMFPFDPAGLDAPDKR | 88 | 110 |
| 817.3952 | 2449.1638 | 2449.1584 | 87 | TGESGVLNMFPFDPAGLDAPDKR | 88 | 110 |
| 817.3952 | 2449.1638 | 2449.1584 | 74 | TGESGVLNMFPFDPAGLDAPDKR | 88 | 110 |
| 817.3953 | 2449.1641 | 2449.1584 | 56 | TGESGVLNMFPFDPAGLDAPDKR | 88 | 110 |
| 817.3957 | 2449.1652 | 2449.1584 | 61 | TGESGVLNMFPFDPAGLDAPDKR | 88 | 110 |
| 817.3975 | 2449.1707 | 2449.1584 | 58 | TGESGVLNMFPFDPAGLDAPDKR | 88 | 110 |
| 548.9505 | 1643.8297 | 1643.8308 | 35 | TLGDGKEEFRPIPW | 182 | 195 |
| 822.9235 | 1643.8324 | 1643.8308 | 61 | TLGDGKEEFRPIPW | 182 | 195 |

| Lhcp1 | | | | | | |
| --- | --- | --- | --- | --- | --- | --- |
| Observed | Mr(expt) | Mr(calc) | Score | Peptide sequence | Amino acid position  start | Amino acid position  end |
| 744.7024 | 2231.0853 | 2231.0859 | 73 | TGLFENPFPELTQGDVTPGGR | 162 | 182 |
| 1116.5503 | 2231.086 | 2231.0859 | 98 | TGLFENPFPELTQGDVTPGGR | 162 | 182 |
| 744.7033 | 2231.0881 | 2231.0859 | 41 | TGLFENPFPELTQGDVTPGGR | 162 | 182 |
| 1116.5516 | 2231.0887 | 2231.0859 | 71 | TGLFENPFPELTQGDVTPGGR | 162 | 182 |
| 1116.5519 | 2231.0892 | 2231.0859 | 82 | TGLFENPFPELTQGDVTPGGR | 162 | 182 |
| 744.7037 | 2231.0894 | 2231.0859 | 46 | TGLFENPFPELTQGDVTPGGR | 162 | 182 |
| 1116.552 | 2231.0894 | 2231.0859 | 23 | TGLFENPFPELTQGDVTPGGR | 162 | 182 |
| 875.9307 | 1749.8468 | 1749.8461 | 77 | FDPLGFAEAGDLEELK | 183 | 198 |

| Lhcap2 | | | | | | |
| --- | --- | --- | --- | --- | --- | --- |
| Observed | Mr(expt) | Mr(calc) | Score | Peptide sequence | Amino acid position  start | Amino acid position  end |
| 590.8121 | 1179.6097 | 1179.6108 | 48 | NAEREVIHGR | 57 | 66 |
| 394.2111 | 1179.6114 | 1179.6108 | 34 | NAEREVIHGR | 57 | 66 |
| 726.0088 | 2175.0045 | 2175.0081 | 53 | TGISDSPFDDGLTVGDVNPGGR | 141 | 162 |
| 726.0089 | 2175.0049 | 2175.0081 | 79 | TGISDSPFDDGLTVGDVNPGGR | 141 | 162 |
| 726.009 | 2175.0051 | 2175.0081 | 52 | TGISDSPFDDGLTVGDVNPGGR | 141 | 162 |
| 726.009 | 2175.0053 | 2175.0081 | 60 | TGISDSPFDDGLTVGDVNPGGR | 141 | 162 |
| 726.0092 | 2175.0057 | 2175.0081 | 61 | TGISDSPFDDGLTVGDVNPGGR | 141 | 162 |
| 726.0095 | 2175.0066 | 2175.0081 | 66 | TGISDSPFDDGLTVGDVNPGGR | 141 | 162 |
| 726.0095 | 2175.0066 | 2175.0081 | 63 | TGISDSPFDDGLTVGDVNPGGR | 141 | 162 |
| 726.0096 | 2175.0069 | 2175.0081 | 62 | TGISDSPFDDGLTVGDVNPGGR | 141 | 162 |
| 1088.5107 | 2175.0069 | 2175.0081 | 90 | TGISDSPFDDGLTVGDVNPGGR | 141 | 162 |
| 1088.511 | 2175.0074 | 2175.0081 | 82 | TGISDSPFDDGLTVGDVNPGGR | 141 | 162 |
| 726.0098 | 2175.0075 | 2175.0081 | 69 | TGISDSPFDDGLTVGDVNPGGR | 141 | 162 |
| 726.0098 | 2175.0077 | 2175.0081 | 56 | TGISDSPFDDGLTVGDVNPGGR | 141 | 162 |
| 1088.5112 | 2175.0079 | 2175.0081 | 94 | TGISDSPFDDGLTVGDVNPGGR | 141 | 162 |
| 1088.5114 | 2175.0081 | 2175.0081 | 134 | TGISDSPFDDGLTVGDVNPGGR | 141 | 162 |
| 726.01 | 2175.0082 | 2175.0081 | 77 | TGISDSPFDDGLTVGDVNPGGR | 141 | 162 |
| 726.01 | 2175.0082 | 2175.0081 | 46 | TGISDSPFDDGLTVGDVNPGGR | 141 | 162 |
| 726.0101 | 2175.0084 | 2175.0081 | 37 | TGISDSPFDDGLTVGDVNPGGR | 141 | 162 |
| 1088.5116 | 2175.0086 | 2175.0081 | 95 | TGISDSPFDDGLTVGDVNPGGR | 141 | 162 |
| 1088.5117 | 2175.0089 | 2175.0081 | 122 | TGISDSPFDDGLTVGDVNPGGR | 141 | 162 |
| 1088.5117 | 2175.0089 | 2175.0081 | 96 | TGISDSPFDDGLTVGDVNPGGR | 141 | 162 |
| 1088.5117 | 2175.0089 | 2175.0081 | 110 | TGISDSPFDDGLTVGDVNPGGR | 141 | 162 |
| 726.0102 | 2175.0089 | 2175.0081 | 70 | TGISDSPFDDGLTVGDVNPGGR | 141 | 162 |
| 726.0102 | 2175.0089 | 2175.0081 | 62 | TGISDSPFDDGLTVGDVNPGGR | 141 | 162 |
| 1088.5118 | 2175.0091 | 2175.0081 | 85 | TGISDSPFDDGLTVGDVNPGGR | 141 | 162 |
| 1088.5118 | 2175.0091 | 2175.0081 | 76 | TGISDSPFDDGLTVGDVNPGGR | 141 | 162 |
| 1088.5118 | 2175.0091 | 2175.0081 | 98 | TGISDSPFDDGLTVGDVNPGGR | 141 | 162 |
| 726.0104 | 2175.0093 | 2175.0081 | 59 | TGISDSPFDDGLTVGDVNPGGR | 141 | 162 |
| 1088.512 | 2175.0094 | 2175.0081 | 97 | TGISDSPFDDGLTVGDVNPGGR | 141 | 162 |
| 1088.512 | 2175.0094 | 2175.0081 | 90 | TGISDSPFDDGLTVGDVNPGGR | 141 | 162 |
| 1088.512 | 2175.0094 | 2175.0081 | 90 | TGISDSPFDDGLTVGDVNPGGR | 141 | 162 |
| 1088.512 | 2175.0094 | 2175.0081 | 103 | TGISDSPFDDGLTVGDVNPGGR | 141 | 162 |
| 726.0104 | 2175.0095 | 2175.0081 | 54 | TGISDSPFDDGLTVGDVNPGGR | 141 | 162 |
| 726.0105 | 2175.0097 | 2175.0081 | 42 | TGISDSPFDDGLTVGDVNPGGR | 141 | 162 |
| 726.0105 | 2175.0097 | 2175.0081 | 22 | TGISDSPFDDGLTVGDVNPGGR | 141 | 162 |
| 726.0106 | 2175.0099 | 2175.0081 | 41 | TGISDSPFDDGLTVGDVNPGGR | 141 | 162 |
| 726.0106 | 2175.0099 | 2175.0081 | 80 | TGISDSPFDDGLTVGDVNPGGR | 141 | 162 |
| 1088.5122 | 2175.0099 | 2175.0081 | 86 | TGISDSPFDDGLTVGDVNPGGR | 141 | 162 |
| 726.0106 | 2175.01 | 2175.0081 | 78 | TGISDSPFDDGLTVGDVNPGGR | 141 | 162 |
| 1088.5123 | 2175.0101 | 2175.0081 | 90 | TGISDSPFDDGLTVGDVNPGGR | 141 | 162 |
| 1088.5123 | 2175.0101 | 2175.0081 | 89 | TGISDSPFDDGLTVGDVNPGGR | 141 | 162 |
| 1088.5124 | 2175.0103 | 2175.0081 | 97 | TGISDSPFDDGLTVGDVNPGGR | 141 | 162 |
| 1088.5124 | 2175.0103 | 2175.0081 | 108 | TGISDSPFDDGLTVGDVNPGGR | 141 | 162 |
| 1088.5124 | 2175.0103 | 2175.0081 | 99 | TGISDSPFDDGLTVGDVNPGGR | 141 | 162 |
| 1088.5124 | 2175.0103 | 2175.0081 | 51 | TGISDSPFDDGLTVGDVNPGGR | 141 | 162 |
| 726.0107 | 2175.0104 | 2175.0081 | 74 | TGISDSPFDDGLTVGDVNPGGR | 141 | 162 |
| 726.0108 | 2175.0106 | 2175.0081 | 54 | TGISDSPFDDGLTVGDVNPGGR | 141 | 162 |
| 1088.5126 | 2175.0106 | 2175.0081 | 80 | TGISDSPFDDGLTVGDVNPGGR | 141 | 162 |
| 726.0109 | 2175.011 | 2175.0081 | 57 | TGISDSPFDDGLTVGDVNPGGR | 141 | 162 |
| 726.011 | 2175.0111 | 2175.0081 | 56 | TGISDSPFDDGLTVGDVNPGGR | 141 | 162 |
| 726.011 | 2175.0113 | 2175.0081 | 60 | TGISDSPFDDGLTVGDVNPGGR | 141 | 162 |
| 1088.5129 | 2175.0113 | 2175.0081 | 97 | TGISDSPFDDGLTVGDVNPGGR | 141 | 162 |
| 1088.5131 | 2175.0116 | 2175.0081 | 90 | TGISDSPFDDGLTVGDVNPGGR | 141 | 162 |
| 1088.5131 | 2175.0116 | 2175.0081 | 106 | TGISDSPFDDGLTVGDVNPGGR | 141 | 162 |
| 866.9363 | 1731.8581 | 1731.8567 | 86 | FDPLGLAESGDLEELK | 163 | 178 |
| 987.5253 | 1973.0361 | 1973.0357 | 40 | FDPLGLAESGDLEELKIK | 163 | 180 |
| 658.6868 | 1973.0385 | 1973.0357 | 28 | FDPLGLAESGDLEELKIK | 163 | 180 |

**Supplementary File 1b. Polypeptides in the A3 fraction (PSI-LHCI supercomplex) as identified by MS analysis.** The bands corresponding to Lhca5 and Lhca6 (Band 2 and 1, respectively) were excised from the SDS-PAGE gel and subsequently subjected to LC-MS/MS analysis following in-gel trypsin digestion, as described in Kubota-Kawai et al. (2019). Detected peptides were analyzed as in Supplementary File 1a.

1. **Band 1**

| **Lhca1** | | | | | | |
| --- | --- | --- | --- | --- | --- | --- |
| Observed | Mr(expt) | Mr(calc) | Score | Peptide sequence | Amino acid position  start | Amino acid position  end |
| 638.3142 | 1274.6137 | 1274.6189 | 27 | YREAELMHAR | 69 | 78 |
| 425.8807 | 1274.6204 | 1274.6189 | 32 | YREAELMHAR | 69 | 78 |
| 431.2114 | 1290.6123 | 1290.6139 | 31 | YREAELMHAR | 69 | 78 |
| 646.3146 | 1290.6147 | 1290.6139 | 45 | YREAELMHAR | 69 | 78 |
| 478.7351 | 955.4556 | 955.4545 | 49 | EAELMHAR | 71 | 78 |
| 486.7324 | 971.4502 | 971.4494 | 52 | EAELMHAR | 71 | 78 |
| 731.7005 | 2192.0797 | 2192.0862 | 42 | QAATGEARLYPGGSFDPAGLSK | 140 | 161 |
| 704.8526 | 1407.6906 | 1407.7034 | 71 | LYPGGSFDPAGLSK | 148 | 161 |
| 704.8555 | 1407.6964 | 1407.7034 | 49 | LYPGGSFDPAGLSK | 148 | 161 |
| 704.8557 | 1407.6969 | 1407.7034 | 49 | LYPGGSFDPAGLSK | 148 | 161 |
| 704.8558 | 1407.697 | 1407.7034 | 50 | LYPGGSFDPAGLSK | 148 | 161 |
| 704.8559 | 1407.6972 | 1407.7034 | 74 | LYPGGSFDPAGLSK | 148 | 161 |
| 704.856 | 1407.6974 | 1407.7034 | 59 | LYPGGSFDPAGLSK | 148 | 161 |
| 704.8563 | 1407.698 | 1407.7034 | 68 | LYPGGSFDPAGLSK | 148 | 161 |
| 704.8588 | 1407.7031 | 1407.7034 | 29 | LYPGGSFDPAGLSK | 148 | 161 |
| 704.8589 | 1407.7032 | 1407.7034 | 42 | LYPGGSFDPAGLSK | 148 | 161 |
| 704.8595 | 1407.7044 | 1407.7034 | 61 | LYPGGSFDPAGLSK | 148 | 161 |
| 704.8595 | 1407.7044 | 1407.7034 | 49 | LYPGGSFDPAGLSK | 148 | 161 |
| 704.8597 | 1407.7048 | 1407.7034 | 73 | LYPGGSFDPAGLSK | 148 | 161 |
| 704.8598 | 1407.705 | 1407.7034 | 42 | LYPGGSFDPAGLSK | 148 | 161 |
| 469.2534 | 936.4923 | 936.4916 | 48 | GKDFETLK | 162 | 169 |
| 469.2534 | 936.4923 | 936.4916 | 22 | GKDFETLK | 162 | 169 |
| 365.205 | 1092.5932 | 1092.5927 | 48 | GKDFETLKR | 162 | 170 |
| 547.3041 | 1092.5936 | 1092.5927 | 52 | GKDFETLKR | 162 | 170 |
| 376.6953 | 751.376 | 751.3752 | 40 | DFETLK | 164 | 169 |
| 845.7332 | 2534.1778 | 2534.1756 | 54 | VAMMAFFGIMAQHQADPSGPGPVK | 178 | 201 |

| **Lhca2** | | | | | | |
| --- | --- | --- | --- | --- | --- | --- |
| Observed | Mr(expt) | Mr(calc) | Score | Peptide sequence | Amino acid position  start | Amino acid position  end |
| 581.6556 | 1741.9449 | 1741.9437 | 42 | WAMLGVAGAVAPELLTK | 84 | 100 |
| 481.769 | 961.5235 | 961.5233 | 65 | LGYAKDPAK | 176 | 184 |
| 481.7691 | 961.5237 | 961.5233 | 50 | LGYAKDPAK | 176 | 184 |
| 481.7692 | 961.5238 | 961.5233 | 56 | LGYAKDPAK | 176 | 184 |
| 481.7693 | 961.524 | 961.5233 | 43 | LGYAKDPAK | 176 | 184 |
| 481.7693 | 961.524 | 961.5233 | 48 | LGYAKDPAK | 176 | 184 |
| 481.7693 | 961.524 | 961.5233 | 43 | LGYAKDPAK | 176 | 184 |
| 481.7693 | 961.5241 | 961.5233 | 73 | LGYAKDPAK | 176 | 184 |
| 481.7693 | 961.5241 | 961.5233 | 70 | LGYAKDPAK | 176 | 184 |
| 481.7694 | 961.5241 | 961.5233 | 32 | LGYAKDPAK | 176 | 184 |
| 481.7694 | 961.5241 | 961.5233 | 57 | LGYAKDPAK | 176 | 184 |
| 481.7696 | 961.5247 | 961.5233 | 44 | LGYAKDPAK | 176 | 184 |
| 581.3344 | 1160.6543 | 1160.6553 | 39 | LGYAKDPAKAK | 176 | 186 |
| 387.8925 | 1160.6556 | 1160.6553 | 30 | LGYAKDPAKAK | 176 | 186 |
| 387.8926 | 1160.6561 | 1160.6553 | 23 | LGYAKDPAKAK | 176 | 186 |
| 581.3353 | 1160.6561 | 1160.6553 | 31 | LGYAKDPAKAK | 176 | 186 |
| 387.8927 | 1160.6562 | 1160.6553 | 42 | LGYAKDPAKAK | 176 | 186 |
| 387.8928 | 1160.6565 | 1160.6553 | 37 | LGYAKDPAKAK | 176 | 186 |
| 387.8928 | 1160.6565 | 1160.6553 | 36 | LGYAKDPAKAK | 176 | 186 |
| 387.8928 | 1160.6566 | 1160.6553 | 41 | LGYAKDPAKAK | 176 | 186 |
| 581.3356 | 1160.6567 | 1160.6553 | 43 | LGYAKDPAKAK | 176 | 186 |
| 387.893 | 1160.6571 | 1160.6553 | 40 | LGYAKDPAKAK | 176 | 186 |
| 387.893 | 1160.6571 | 1160.6553 | 29 | LGYAKDPAKAK | 176 | 186 |
| 500.2958 | 998.5771 | 998.576 | 50 | DPAKAKELK | 181 | 189 |
| 500.8242 | 999.6339 | 999.6328 | 34 | ELKLKEIK | 187 | 194 |

| **Lhca4** | | | | | | |
| --- | --- | --- | --- | --- | --- | --- |
| Observed | Mr(expt) | Mr(calc) | Score | Peptide sequence | Amino acid position  start | Amino acid position  end |
| 691.986 | 2072.9362 | 2072.9448 | 47 | ASTQPMWFPGMDAPQHLK | 30 | 47 |
| 691.9894 | 2072.9465 | 2072.9448 | 35 | ASTQPMWFPGMDAPQHLK | 30 | 47 |
| 846.4179 | 1690.8213 | 1690.8203 | 49 | GELPGDYGFDPLNLGK | 48 | 63 |
| 857.9636 | 1713.9127 | 1713.9124 | 96 | WAMLGVAGAAAPEILTK | 81 | 97 |
| 572.3119 | 1713.9138 | 1713.9124 | 22 | WAMLGVAGAAAPEILTK | 81 | 97 |
| 368.1656 | 734.3166 | 734.317 | 22 | WQDMR | 138 | 142 |
| 368.166 | 734.3175 | 734.317 | 22 | WQDMR | 138 | 142 |
| 570.5161 | 2278.0353 | 2278.0437 | 52 | WQDMRKPGSVNEDPAFSGNK | 138 | 157 |
| 516.2552 | 1545.7437 | 1545.7423 | 34 | KPGSVNEDPAFSGNK | 143 | 157 |
| 394.2376 | 786.4606 | 786.4599 | 38 | GDLNKLK | 178 | 184 |
| 394.2376 | 786.4607 | 786.4599 | 36 | GDLNKLK | 178 | 184 |
| 394.2378 | 786.461 | 786.4599 | 45 | GDLNKLK | 178 | 184 |
| 493.8039 | 985.5933 | 985.592 | 39 | GDLNKLKAK | 178 | 186 |

| **PsbO** | | | | | | |
| --- | --- | --- | --- | --- | --- | --- |
| Observed | Mr(expt) | Mr(calc) | Score | Peptide sequence | Amino acid position  start | Amino acid position  end |
| 739.3484 | 1476.6822 | 1476.6807 | 40 | LTYTLDGMTGDFK | 138 | 150 |
| 865.9107 | 1729.8069 | 1729.806 | 46 | GNTSQFGGDFVVPSYR | 193 | 208 |
| 425.7194 | 849.4242 | 849.4232 | 33 | GSSFLDPK | 209 | 216 |
| 554.6172 | 1660.8299 | 1660.8281 | 51 | GRGGSTGYDNAVALPAR | 217 | 233 |
| 724.8602 | 1447.7058 | 1447.7056 | 95 | GGSTGYDNAVALPAR | 219 | 233 |
| 618.3433 | 1234.6721 | 1234.671 | 63 | ITGLWYGNIAK | 292 | 302 |

| **PsaC** | | | | | | |
| --- | --- | --- | --- | --- | --- | --- |
| Observed | Mr(expt) | Mr(calc) | Score | Peptide sequence | Amino acid position  start | Amino acid position  end |
| 823.3607 | 1644.7068 | 1644.7059 | 76 | IYDTCIGCTQCVR | 7 | 19 |
| 435.7432 | 869.4719 | 869.4719 | 85 | AGQIASAPR | 36 | 44 |
| 375.4995 | 1123.4768 | 1123.475 | 23 | TEDCVGCKR | 45 | 53 |
| 763.3432 | 1524.6718 | 1524.6701 | 72 | CEAACPTDFLSVR | 54 | 66 |

| **Lhcb4** | | | | | | |
| --- | --- | --- | --- | --- | --- | --- |
| Observed | Mr(expt) | Mr(calc) | Score | Peptide sequence | Amino acid position  start | Amino acid position  end |
| 793.3785 | 1584.7424 | 1584.7494 | 64 | TIVPYDEAFDIMR | 109 | 121 |
| 530.7733 | 1059.5321 | 1059.5309 | 53 | SAELDQNKR | 192 | 200 |
| 801.3837 | 1600.7529 | 1600.7596 | 62 | MYPGGAFDPLGYVAK | 201 | 215 |
| 603.8098 | 1205.6049 | 1205.604 | 48 | ADAQEEFRLK | 216 | 225 |

| **Lhca6** | | | | | | |
| --- | --- | --- | --- | --- | --- | --- |
| Observed | Mr(expt) | Mr(calc) | Score | Peptide sequence | Amino acid position  start | Amino acid position  end |
| 352.2182 | 702.4219 | 702.4211 | 24 | VRGLMK | 82 | 87 |
| 817.3898 | 2449.1477 | 2449.1584 | 70 | TGESGVLNMFPFDPAGLDAPDKR | 88 | 110 |
| 548.9505 | 1643.8297 | 1643.8308 | 30 | TLGDGKEEFRPIPW | 182 | 195 |
| 822.9231 | 1643.8316 | 1643.8308 | 70 | TLGDGKEEFRPIPW | 182 | 195 |
| 537.274 | 1072.5335 | 1072.5342 | 36 | EEFRPIPW | 188 | 195 |

1. **Band 2**

| **PsaD** | | | | | | |
| --- | --- | --- | --- | --- | --- | --- |
| Observed | Mr(expt) | Mr(calc) | Score | Peptide sequence | Amino acid position  start | Amino acid position  end |
| 514.7717 | 2055.0576 | 2055.0636 | 31 | AEEAKAKTESAAPAVWTAPK | 31 | 50 |
| 686.0275 | 2055.0606 | 2055.0636 | 67 | AEEAKAKTESAAPAVWTAPK | 31 | 50 |
| 514.7726 | 2055.0612 | 2055.0636 | 34 | AEEAKAKTESAAPAVWTAPK | 31 | 50 |
| 514.7731 | 2055.0632 | 2055.0636 | 41 | AEEAKAKTESAAPAVWTAPK | 31 | 50 |
| 514.7732 | 2055.0639 | 2055.0636 | 31 | AEEAKAKTESAAPAVWTAPK | 31 | 50 |
| 514.7737 | 2055.0656 | 2055.0636 | 24 | AEEAKAKTESAAPAVWTAPK | 31 | 50 |
| 514.7737 | 2055.0659 | 2055.0636 | 31 | AEEAKAKTESAAPAVWTAPK | 31 | 50 |
| 764.4083 | 1526.8021 | 1526.8093 | 54 | AKTESAAPAVWTAPK | 36 | 50 |
| 509.944 | 1526.8103 | 1526.8093 | 22 | AKTESAAPAVWTAPK | 36 | 50 |
| 509.9441 | 1526.8104 | 1526.8093 | 50 | AKTESAAPAVWTAPK | 36 | 50 |
| 764.4125 | 1526.8104 | 1526.8093 | 85 | AKTESAAPAVWTAPK | 36 | 50 |
| 509.9441 | 1526.8105 | 1526.8093 | 45 | AKTESAAPAVWTAPK | 36 | 50 |
| 509.9441 | 1526.8105 | 1526.8093 | 42 | AKTESAAPAVWTAPK | 36 | 50 |
| 509.9442 | 1526.8106 | 1526.8093 | 49 | AKTESAAPAVWTAPK | 36 | 50 |
| 509.9442 | 1526.8107 | 1526.8093 | 32 | AKTESAAPAVWTAPK | 36 | 50 |
| 509.9445 | 1526.8116 | 1526.8093 | 49 | AKTESAAPAVWTAPK | 36 | 50 |
| 664.8425 | 1327.6704 | 1327.6772 | 24 | TESAAPAVWTAPK | 38 | 50 |
| 664.8435 | 1327.6725 | 1327.6772 | 37 | TESAAPAVWTAPK | 38 | 50 |
| 664.8437 | 1327.6728 | 1327.6772 | 78 | TESAAPAVWTAPK | 38 | 50 |
| 664.8461 | 1327.6776 | 1327.6772 | 50 | TESAAPAVWTAPK | 38 | 50 |
| 664.8462 | 1327.6778 | 1327.6772 | 66 | TESAAPAVWTAPK | 38 | 50 |
| 664.8464 | 1327.6782 | 1327.6772 | 69 | TESAAPAVWTAPK | 38 | 50 |
| 664.8464 | 1327.6782 | 1327.6772 | 69 | TESAAPAVWTAPK | 38 | 50 |
| 664.8467 | 1327.6788 | 1327.6772 | 64 | TESAAPAVWTAPK | 38 | 50 |
| 664.847 | 1327.6794 | 1327.6772 | 54 | TESAAPAVWTAPK | 38 | 50 |
| 633.6706 | 1897.9901 | 1897.9898 | 62 | LDPNTPSPIFGGSTGGLLR | 51 | 69 |
| 574.6271 | 1720.8596 | 1720.864 | 25 | KEAIFEMPTGGAAIMR | 85 | 100 |
| 579.9609 | 1736.8608 | 1736.8589 | 39 | KEAIFEMPTGGAAIMR | 85 | 100 |
| 585.2924 | 1752.8553 | 1752.8539 | 40 | KEAIFEMPTGGAAIMR | 85 | 100 |
| 627.9905 | 1880.9498 | 1880.9488 | 26 | KEAIFEMPTGGAAIMRK | 85 | 101 |
| 805.3856 | 1608.7566 | 1608.764 | 92 | EAIFEMPTGGAAIMR | 86 | 100 |
| 805.3865 | 1608.7584 | 1608.764 | 36 | EAIFEMPTGGAAIMR | 86 | 100 |
| 805.3869 | 1608.7592 | 1608.764 | 21 | EAIFEMPTGGAAIMR | 86 | 100 |
| 813.3832 | 1624.7519 | 1624.7589 | 22 | EAIFEMPTGGAAIMR | 86 | 100 |
| 813.387 | 1624.7595 | 1624.7589 | 23 | EAIFEMPTGGAAIMR | 86 | 100 |
| 542.5942 | 1624.7607 | 1624.7589 | 46 | EAIFEMPTGGAAIMR | 86 | 100 |
| 813.3877 | 1624.7608 | 1624.7589 | 72 | EAIFEMPTGGAAIMR | 86 | 100 |
| 579.9581 | 1736.8524 | 1736.8589 | 28 | EAIFEMPTGGAAIMRK | 86 | 101 |
| 385.2499 | 768.4852 | 768.4857 | 39 | KGPNLLK | 101 | 107 |
| 385.2505 | 768.4864 | 768.4857 | 22 | KGPNLLK | 101 | 107 |
| 385.2507 | 768.4869 | 768.4857 | 32 | KGPNLLK | 101 | 107 |
| 385.2508 | 768.4871 | 768.4857 | 21 | KGPNLLK | 101 | 107 |
| 385.2509 | 768.4873 | 768.4857 | 18 | KGPNLLK | 101 | 107 |
| 491.3142 | 980.6139 | 980.6131 | 53 | GPNLLKLAR | 102 | 110 |
| 746.8978 | 1491.7811 | 1491.7868 | 74 | KEQCLALLNTFR | 111 | 122 |
| 498.2695 | 1491.7867 | 1491.7868 | 41 | KEQCLALLNTFR | 111 | 122 |
| 569.9792 | 1706.9159 | 1706.9137 | 18 | KEQCLALLNTFRSK | 111 | 124 |
| 682.8536 | 1363.6926 | 1363.6918 | 63 | EQCLALLNTFR | 112 | 122 |
| 578.2867 | 1154.5589 | 1154.5576 | 36 | MKLDGCIYR | 125 | 133 |
| 385.8604 | 1154.5592 | 1154.5576 | 36 | MKLDGCIYR | 125 | 133 |
| 586.284 | 1170.5534 | 1170.5525 | 52 | MKLDGCIYR | 125 | 133 |
| 500.9333 | 1499.778 | 1499.7773 | 32 | VFPSGEVQYLHPK | 134 | 146 |
| 750.8966 | 1499.7785 | 1499.7773 | 66 | VFPSGEVQYLHPK | 134 | 146 |
| 500.9336 | 1499.779 | 1499.7773 | 36 | VFPSGEVQYLHPK | 134 | 146 |
| 750.8969 | 1499.7793 | 1499.7773 | 63 | VFPSGEVQYLHPK | 134 | 146 |
| 404.1981 | 806.3817 | 806.381 | 31 | DGVYPEK | 147 | 153 |
| 383.5364 | 1147.5872 | 1147.5873 | 28 | DGVYPEKVNK | 147 | 156 |
| 574.8011 | 1147.5876 | 1147.5873 | 43 | DGVYPEKVNK | 147 | 156 |
| 574.8013 | 1147.588 | 1147.5873 | 20 | DGVYPEKVNK | 147 | 156 |
| 454.5769 | 1360.709 | 1360.7099 | 47 | DGVYPEKVNKGR | 147 | 158 |
| 681.363 | 1360.7115 | 1360.7099 | 45 | DGVYPEKVNKGR | 147 | 158 |
| 368.1897 | 1101.5472 | 1101.5461 | 30 | GRVGANQNMR | 157 | 166 |
| 551.7809 | 1101.5473 | 1101.5461 | 56 | GRVGANQNMR | 157 | 166 |
| 559.7773 | 1117.5401 | 1117.5411 | 48 | GRVGANQNMR | 157 | 166 |
| 373.5212 | 1117.5418 | 1117.5411 | 29 | GRVGANQNMR | 157 | 166 |
| 445.2174 | 888.4203 | 888.4236 | 63 | VGANQNMR | 159 | 166 |
| 453.2167 | 904.4189 | 904.4185 | 27 | VGANQNMR | 159 | 166 |
| 453.217 | 904.4194 | 904.4185 | 50 | VGANQNMR | 159 | 166 |
| 453.2171 | 904.4197 | 904.4185 | 50 | VGANQNMR | 159 | 166 |
| 453.2207 | 904.4269 | 904.4185 | 35 | VGANQNMR | 159 | 166 |
| 639.3309 | 1914.9708 | 1914.9694 | 31 | VGANQNMRSIGKNTNPAK | 159 | 176 |
| 515.2857 | 1028.5569 | 1028.5614 | 49 | SIGKNTNPAK | 167 | 176 |
| 515.2886 | 1028.5626 | 1028.5614 | 49 | SIGKNTNPAK | 167 | 176 |
| 515.2887 | 1028.5628 | 1028.5614 | 37 | SIGKNTNPAK | 167 | 176 |
| 635.8752 | 1269.7358 | 1269.7404 | 54 | SIGKNTNPAKIK | 167 | 178 |
| 635.8757 | 1269.7369 | 1269.7404 | 16 | SIGKNTNPAKIK | 167 | 178 |
| 424.2547 | 1269.7423 | 1269.7404 | 24 | SIGKNTNPAKIK | 167 | 178 |
| 360.7234 | 719.4323 | 719.433 | 29 | IKFQGK | 177 | 182 |
| 561.3036 | 1120.5926 | 1120.5917 | 49 | FQGKLGPFE | 179 | 188 |

| **PsaF** | | | | | | |
| --- | --- | --- | --- | --- | --- | --- |
| Observed | Mr(expt) | Mr(calc) | Score | Peptide sequence | Amino acid position  start | Amino acid position  end |
| 466.277 | 930.5393 | 930.5386 | 22 | TELKALEK | 90 | 97 |
| 363.2211 | 1086.6414 | 1086.6397 | 25 | TELKALEKR | 90 | 98 |
| 740.7252 | 2219.1537 | 2219.1507 | 74 | LKKYDPESAPALALQATMEK | 99 | 118 |
| 555.7958 | 2219.1543 | 2219.1507 | 44 | LKKYDPESAPALALQATMEK | 99 | 118 |
| 655.0005 | 1961.9796 | 1961.9768 | 41 | KYDPESAPALALQATMEK | 101 | 118 |
| 660.3313 | 1977.9721 | 1977.9717 | 36 | KYDPESAPALALQATMEK | 101 | 118 |
| 660.3315 | 1977.9726 | 1977.9717 | 53 | KYDPESAPALALQATMEK | 101 | 118 |
| 660.3318 | 1977.9737 | 1977.9717 | 50 | KYDPESAPALALQATMEK | 101 | 118 |
| 660.3326 | 1977.9759 | 1977.9717 | 27 | KYDPESAPALALQATMEK | 101 | 118 |
| 736.7126 | 2207.1159 | 2207.1143 | 74 | KYDPESAPALALQATMEKTK | 101 | 120 |
| 552.7864 | 2207.1164 | 2207.1143 | 26 | KYDPESAPALALQATMEKTK | 101 | 120 |
| 617.6335 | 1849.8788 | 1849.8767 | 47 | YDPESAPALALQATMEK | 102 | 118 |
| 708.3324 | 1414.6502 | 1414.6551 | 79 | FANYGESGLLCGK | 123 | 135 |
| 441.2217 | 880.4289 | 880.429 | 39 | SYIQENK | 173 | 179 |
| 640.8439 | 2559.3464 | 2559.3432 | 45 | SYIQENKTASKPTEGEIIIDVPK | 173 | 195 |
| 566.6474 | 1696.9204 | 1696.9247 | 24 | TASKPTEGEIIIDVPK | 180 | 195 |
| 849.4701 | 1696.9256 | 1696.9247 | 66 | TASKPTEGEIIIDVPK | 180 | 195 |
| 566.6493 | 1696.926 | 1696.9247 | 46 | TASKPTEGEIIIDVPK | 180 | 195 |
| 836.4316 | 1670.8486 | 1670.8475 | 73 | NGTLTAPESEITVSPR | 216 | 231 |
| 557.9573 | 1670.85 | 1670.8475 | 59 | NGTLTAPESEITVSPR | 216 | 231 |
| 836.4323 | 1670.8501 | 1670.8475 | 65 | NGTLTAPESEITVSPR | 216 | 231 |

| **Lhca5** | | | | | | |
| --- | --- | --- | --- | --- | --- | --- |
| Observed | Mr(expt) | Mr(calc) | Score | Peptide sequence | Amino acid position  start | Amino acid position  end |
| 575.3109 | 1148.6073 | 1148.6052 | 28 | KMWLPAPYK | 32 | 40 |
| 511.2625 | 1020.5105 | 1020.5103 | 27 | MWLPAPYK | 33 | 40 |
| 543.6088 | 1627.8047 | 1627.8028 | 41 | LKYYQEAELMNAR | 67 | 79 |
| 548.9404 | 1643.7995 | 1643.7977 | 53 | LKYYQEAELMNAR | 67 | 79 |
| 694.3185 | 1386.6224 | 1386.6237 | 76 | YYQEAELMNAR | 69 | 79 |
| 694.3201 | 1386.6256 | 1386.6237 | 25 | YYQEAELMNAR | 69 | 79 |
| 702.3142 | 1402.6137 | 1402.6187 | 33 | YYQEAELMNAR | 69 | 79 |
| 702.3149 | 1402.6152 | 1402.6187 | 55 | YYQEAELMNAR | 69 | 79 |
| 702.3157 | 1402.6168 | 1402.6187 | 62 | YYQEAELMNAR | 69 | 79 |
| 468.5472 | 1402.6197 | 1402.6187 | 45 | YYQEAELMNAR | 69 | 79 |
| 702.3175 | 1402.6204 | 1402.6187 | 52 | YYQEAELMNAR | 69 | 79 |
| 410.2238 | 818.4331 | 818.432 | 38 | DANMKLK | 157 | 163 |

| **PsaC** | | | | | | |
| --- | --- | --- | --- | --- | --- | --- |
| Observed | Mr(expt) | Mr(calc) | Score | Peptide sequence | Amino acid position  start | Amino acid position  end |
| 823.3613 | 1644.708 | 1644.7059 | 38 | IYDTCIGCTQCVR | 7 | 19 |
| 435.7435 | 869.4725 | 869.4719 | 96 | AGQIASAPR | 36 | 44 |
| 375.4997 | 1123.4772 | 1123.475 | 20 | TEDCVGCKR | 45 | 53 |
| 763.3393 | 1524.664 | 1524.6701 | 48 | CEAACPTDFLSVR | 54 | 66 |
